# The effect of self-limiting on the prevention and control of the diffuse COVID-19 epidemic with delayed and temporal-spatial heterogeneous

**DOI:** 10.1186/s12879-021-06670-y

**Published:** 2021-11-09

**Authors:** Cheng-Cheng Zhu, Jiang Zhu

**Affiliations:** 1 School of Science, Jiangnan University, Wuxi, 214122 China; 2 School of Mathematics and Statistics, Jiangsu Normal University, Xuzhou, 221116 China

**Keywords:** Self-limiting epidemics, COVID-19, Global exponential attractor, Delayed, Temporal-spatial heterogeneous

## Abstract

**Background:**

The global spread of the novel coronavirus pneumonia is still continuing, and a new round of more serious outbreaks has even begun in some countries. In this context, this paper studies the dynamics of a type of delayed reaction-diffusion novel coronavirus pneumonia model with relapse and self-limiting treatment in a temporal-spatial heterogeneous environment.

**Methods:**

First, focus on the self-limiting characteristics of COVID-19, incorporate the relapse and self-limiting treatment factors into the diffusion model, and study the influence of self-limiting treatment on the diffusion of the epidemic. Second, because the traditional Lyapunov stability method is difficult to determine the spread of the epidemic with relapse and self-limiting treatment, we introduce a completely different method, relying on the existence conditions of the exponential attractor of our newly established in the infinite-dimensional dynamic system to determine the diffusion of novel coronavirus pneumonia. Third, relapse and self-limiting treatment have led to a change in the structure of the delayed diffusion COVID-19 model, and the traditional basic reproduction number $R_0$ no longer has threshold characteristics. With the help of the Krein-Rutman theorem and the eigenvalue method, we studied the threshold characteristics of the principal eigenvalue and found that it can be used as a new threshold to describe the diffusion of the epidemic.

**Results:**

Our results prove that the principal eigenvalue $\uplambda ^{*}$ of the delayed reaction-diffusion COVID-19 system with relapse and self-limiting treatment can replace the basic reproduction number $R_0$ to describe the threshold effect of disease transmission. Combine with the latest official data and the prevention and control strategies, some numerical simulations on the stability and global exponential attractiveness of the diffusion of the COVID-19 epidemic in China and the USA are given.

**Conclusions:**

Through the comparison of numerical simulations, we find that self-limiting treatment can significantly promote the prevention and control of the epidemic. And if the free activities of asymptomatic infected persons are not restricted, it will seriously hinder the progress of epidemic prevention and control.

## Background

Many self-limiting diseases are contagious, such as influenza, chickenpox, hepatitis A, acute hepatitis B, Ebola virus and Norovirus are self-limiting epidemics. On February 10, 2020, at the COVID-19 epidemic prevention and control press conference held in Hubei Province, Zhang Dingyu, president of Wuhan Jinyintan Hospital, introduced that novel coronavirus pneumonia is actually a self-limiting disease. As early as 1835, American medical scientist Jacob Bigelow noticed such diseases. In his paper “Self-Limited Diseases”, he pointed out that some diseases exhibits the characteristics of “self-limiting”, and these diseases are limited by their own nature, rather than external influences. Self-limiting disease does not mean that you can stop seeking medical attention. Instead, it reminds the public to maintain adequate rest and adequate nutritional intake to improve immunity, and under the premise of personal protection, there is no need to panic about diseases.

The global outbreak of the novel coronavirus pneumonia has entered a stage of normalization and has not yet been effectively controlled [5–7, 10, 14]. The degree of activity of the new crown virus is highly dependent on temperature. As the northern hemisphere enters a cold winter, the epidemic situation in many countries has shown a momentum of secondary outbreaks. People infected with novel coronavirus pneumonia are now divided into four stages: mild, normal, severe, and critical. People with mild infections can recover quickly with oxygen therapy, symptomatic treatment and immunotherapy while being quarantined at home or in the hospital. Last year, many football and basketball players (such as Zidane, Wu Lei, Gobert) were once infected with a mild new crown virus. Due to their superior physical fitness, they recovered faster than ordinary people after targeted treatment. How to better prevent and control the diffusion of novel coronavirus pneumonia has become a hot spot on the global medical community. Although the theoretical research on the infection of the novel coronavirus pneumonia epidemic has been carried out for more than a year. However, from the current research results, most of the researches are still based on ordinary differential equations. In Algehyne’s study [1], a new mathematical SQIR model for COVID-19 formed by taking into account the impact of quarantine has been examined. Although authors performed a detailed analysis of the local and global stability of the model, but they ignored the huge impact of the exposed population on the infection of the COVID-19 epidemic. The authors of [2] used actual data to study the evolution of fatalities arising from coronavirus COVID-19 worldwide. Bentout et al. [4] forecast the progress of the COVID-19 in the USA, the United Arab Emirates and Algeria by an age-structured model. Shahzad et al. [15] developed the models for coronavirus disease at different stages with the addition of more parameters due to interactions among the individuals. Then, some key computational simulations and sensitivity analysis are investigated. Appadu’s study [3] gave a comparison of some existing forecasting methods about COVID-19, while Das et al. [10] gave a comparison of different intervention strategies for the prevention and control of Corona Virus Disease 2019 epidemic in their article. These results, whether discussing the global stability of the model or predicting the development of the epidemic, ignore the strong dependence of the Corona Virus Disease 2019 epidemic on spatial diffusion, heterogeneous environment and population flow. In 2020, we present a method of global exponential attractor in the reaction-diffusion infectious disease model in spatial heterogeneous environment to study the spread trend and long-term dynamic behavior of the COVID-19 epidemic [22]. In 2021, we study a reaction-diffusion COVID-19 model with home quarantine, standard contact rate, time delay and relapse in the temporal-spatial heterogeneous environment. Except for the diffusion coefficient, other coefficients of this model are temporal-spatial heterogeneous [23].

The novel coronavirus pneumonia epidemic has spread globally for more than a year. China is undoubtedly the country with the most successful epidemic prevention and control among the populous countries. Currently, asymptomatic infections, imported cases, imported cold-chain food packaging and other items that carry the virus are the main sources of new confirmed cases in China. With the intervention of nucleic acid detection methods and the successful development of vaccines, a substantial breakthrough has been made in the global prevention and control of the Corona Virus Disease 2019. Nucleic acid testing is currently the fastest and most effective method to find asymptomatic infections. The injection of vaccines can enhance the immunity, resistance and self-healing ability of susceptible individuals. novel coronavirus pneumonia epidemic is highly dependent on climate, temperature and humidity, and the mobility and density of the population will also affect the spread of the disease. The new crown virus is unusually active in winter and early spring and is prone to large-scale outbreaks. Large-scale personnel gathering and population movement will increase the possibility of infection. Therefore, during the Spring Festival of 2021, the Chinese government advises people to reduce travel and encourages everyone to spend the Lunar New Year where they work. It is known to all that Corona Virus Disease 2019 has an incubation period, and the infected person cannot be detected immediately afterwards. Through the above description, we find that it is necessary to add factors such as self-limiting, temporal-spatial heterogeneous, time delay, asymptomatic infection and virus-carrying items into the model. Considering these factors can make our model more consistent with the diffusion of the Corona Virus Disease 2019. However, the more factors considered, the greater the number of equations in the system, and the greater the coupling relationship between the equations, which makes theoretical research and reasoning more difficult.

Different from the previous results that discussed the dynamics of the infectious disease model, the model in this article has increased the coupling between the equations because of the addition of relapse and self-limiting treatment. Therefore, the basic reproduction number $R_{0}$ commonly used to describe the transmission capacity of infectious diseases is not enough to accurately describe the infection capacity of the novel coronavirus pneumonia epidemic with self-limiting treatment and relapse. At this time, we need to find another parameter with threshold characteristics to measure the infection of Corona Virus Disease 2019. Through theoretical derivation, we find that the principal eigenvalue $\uplambda ^{*}$ of the system has this threshold characteristic. Since we have added self-limiting treatment and temporal-spatial heterogeneous environment to the model, the number of equations in the system has increased and all coefficients are related to the temporal-spatial heterogeneous environment, which greatly increases the technical difficulty of constructing Lyapunov functionals. It is difficult to find a suitable Lyapunov functional to prove the global asymptotic stability of the novel coronavirus pneumonia model with self-limiting treatment in the temporal-spatial heterogeneous environment.

## Methods

### Construction of a model for the diffusion of novel coronavirus pneumonia

**Fig. 1:**
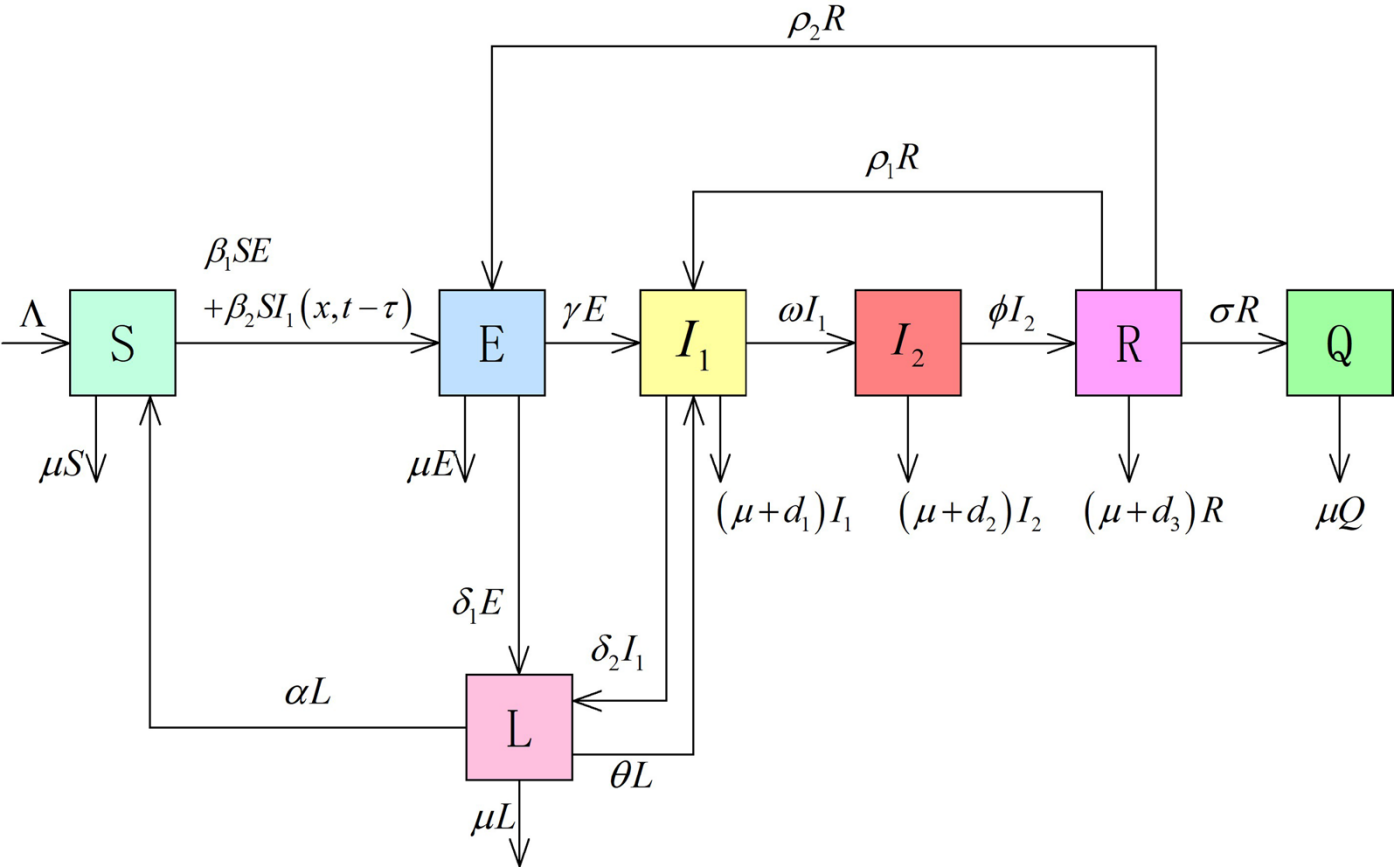
Transfer diagram for the self-limiting novel coronavirus pneumonia epidemics with relapse in temporal-spatial heterogeneous environment

First, we construct a delayed reaction-diffusion and self-limiting novel coronavirus pneumonia epidemic model with relapse and bilinear contact in a temporal-spatial heterogeneous environment. The parameter description and infection mechanism diagram as shown in Table 1 and Fig. 1.

**Table 1:** Parameter description of self-limiting novel coronavirus pneumonia epidemics

Parameter	Description
*S* (*x*, *t*)	Density of susceptible individuals at location x and time t
*E* (*x*, *t*)	Density of patients with incubation period, asymptomatic infections, and items carrying the virus at location x and time t
*L* (*x*, *t*)	Density of individuals undergoing self-limiting treatment at location x and time t
$I_{1}(x,t)$	Density of individuals with mild and common infections at location x and time t
$I_{2}(x,t)$	Density of Severe and high-risk patients in home at location x and time t
*R* (*x*, *t*)	Density of temporary restorers at location x and time t
*Q* (*x*, *t*)	Density of completely cured individuals at location x and time t
$\Lambda (x,t)$	Total recruitment scale into this homogeneous social mixing community at location x and time t
$\beta _{i} (x,t),i=1,2$	Effective contact ratio at location x and time t
$\delta _{i} (x,t),i=1,2$	Self-limiting treatment ratio at location x and time t
$\alpha (x,t)$	Success ratio of the self-limiting treatment at location x and time t
$\theta (x,t)$	Failure ratio self-limiting treatment at location x and time t
$\gamma (x,t)$	Incidence ratio at location x and time t
$\omega (x,t)$	Deterioration rate at location x and time t
$\phi (x,t)$	Temporary recovery ratio at location x and time t
$\rho _{i} (x,t),i=1,2$	Relapse ratio at location x and time t
$\sigma (x,t)$	Complete cure ratio at location x and time t
$\mu (x,t)$	Natural mortality ratio at location x and time t
$\eta _{i} (x,t),i=1,2,3$	Disease-related death ratio at location x and time t
$d_{S}\left( x\right) ,d_{E}\left( x\right) ,d_{L}\left( x\right) ,$	Diffusion ratio at location x
$$d_{I_{1}}\left( x\right) ,d_{R}\left( x\right)$$	

1 $$\begin{aligned} {\left\{ \begin{array}{ll} \frac{\partial S}{\partial t}=\nabla \cdot \left( {d_{S}}\left( x\right) \nabla S\right) +\Lambda \left( x,t\right) -{\beta _{1}}\left( x,t\right) SE-{\beta _{2}}\left( x,t\right) {SI_{1}}\left( x,t-\tau \right) \\ \text { \ \ \ \ \ \ }+\alpha \left( x,t\right) L-\mu \left( x,t\right) S, \\ \frac{\partial E}{\partial t}=\nabla \cdot \left( {d_{E}}\left( x\right) \nabla E\right) +{\beta _{1}}\left( x,t\right) SE+{\beta _{2}}\left( x,t\right) {SI_{1}}\left( x,t-\tau \right) +{\rho _{2}}\left( x,t\right) R \\ \text { \ \ \ \ \ \ }-\left[ {\delta _{1}}\left( x,t\right) +\gamma \left( x,t\right) +\mu \left( x,t\right) \right] E, \\ \frac{\partial L}{\partial t}=\nabla \cdot \left( {d_{L}}\left( x\right) \nabla L\right) +{\delta _{1}}\left( x,t\right) E+{\delta _{2}}\left( x,t\right) I_{1}-\left[ \alpha \left( x,t\right) +\theta \left( x,t\right) +\mu \left( x,t\right) \right] L, \\ \frac{\partial I_{1}}{\partial t}=\nabla \cdot \left( {d_{I_{1}}}\left( x\right) \nabla I_{1}\right) +\gamma \left( x,t\right) E+\theta \left( x,t\right) L+{\rho _{1}}\left( x,t\right) R \\ \text { \ \ \ \ \ \ }-\left[ {\delta _{2}}\left( x,t\right) +\omega \left( x,t\right) +\mu \left( x,t\right) +{\eta _{1}}\left( x,t\right) \right] I_{1}, \\ \frac{\partial I_{2}}{\partial t}=\omega \left( x,t\right) I_{1}-\left[ \phi \left( x,t\right) +\mu \left( x,t\right) +{\eta _{2}}\left( x,t\right) \right] I_{2}, \\ \frac{\partial R}{\partial t}=\nabla \cdot \left( d_{R}\left( x\right) \nabla R\right) +\phi \left( x,t\right) I_{2}-\left[ {\rho _{1}}\left( x,t\right) +{\rho _{2}}\left( x,t\right) +\sigma \left( x,t\right) +\mu \left( x,t\right) +{\eta _{3}}\left( x,t\right) \right] R, \\ \frac{\partial Q}{\partial t}=\sigma \left( x,t\right) R-\mu \left( x,t\right) Q,\text { \ \ \ \ \ \ \ }x\in \Omega ,t>0, \\ \frac{\partial S}{\partial {\mathbf {n}}}=\frac{\partial E}{\partial {\mathbf {n}}} =\frac{\partial L}{\partial {\mathbf {n}}}=\frac{\partial I_{1}}{\partial {\mathbf {n}}}=\frac{\partial I_{2}}{\partial {\mathbf {n}}}=\frac{\partial R}{ \partial {\mathbf {n}}}=\frac{\partial Q}{\partial {\mathbf {n}}}=0,\text { }x\in \partial \Omega ,t>0, \\ S\left( x,s\right) =S_{0}\left( x,s\right) \ge 0,E\left( x,s\right) =E_{0}\left( x,s\right) \ge 0,L\left( x,s\right) =L_{0}\left( x,s\right) \ge 0, \\ I_{1}\left( x,s\right) =I_{10}\left( x,s\right) \ge 0,I_{2}\left( x,s\right) =I_{20}\left( x,s\right) \ge 0,R\left( x,s\right) =R_{0}\left( x,s\right) \ge 0, \\ Q\left( x,s\right) =Q_{0}\left( x,s\right) \ge 0,x\in \Omega ,-\tau \le s\le 0. \end{array}\right. } \end{aligned}$$

Here, $\Omega$ is a bounded domain in ${\mathbb {R}}^{m}\left( m\ge 1\right)$ and the boundary $\partial \Omega$ is smooth, $d_{S}\left( x\right) ,d_{E}\left( x\right) ,d_{L}\left( x\right) , d_{I_{1}}\left( x\right) ,d_{R}\left( x\right) \in {\mathbf {C}}^{1}\left( \Omega \right)$ are the space-dependent positive continuous uniformly bounded diffusion coefficient, ${\Lambda } \left( x,t\right) ,{\beta _{1}}\left( x,t\right) ,{\beta _{2}}\left( x,t\right) , {\rho _{1}}\left( x,t\right) , {\rho _{2}}\left( x,t\right) ,{\alpha }\left( x,t\right) ,{\gamma } \left( x,t\right) ,{\mu }\left( x,t\right) , {\delta _{1}}\left( x,t\right) , {\delta _{2}}\left( x,t\right) , {\gamma } \left( x,t\right) ,{\theta } \left( x,t\right) ,{\omega } \left( x,t\right) ,{\sigma }\left( x,t\right) , {\eta _{1}}\left( x,t\right) ,{\eta _{2}}\left( x,t\right)$ and ${\eta _{3}}\left( x,t\right)$ are positive Hölder continuous functions about the total recruitment scale, rates of contact, relapse, incidence, quarantined, recovery, natural death and disease-related death respectively. $\frac{\partial S}{\partial {\mathbf {n}}}= \frac{\partial E}{\partial {\mathbf {n}}}=\frac{\partial L}{\partial {\mathbf {n}}} =\frac{\partial I_{1}}{\partial {\mathbf {n}}}=\frac{\partial I_{2}}{\partial {\mathbf {n}}}=\frac{\partial R}{\partial {\mathbf {n}}}=\frac{\partial Q}{ \partial {\mathbf {n}}}=0$ denotes that the change ratio on the boundary is equal to 0. ${\beta _{1}}\left( x,t\right) SE$ and ${\beta _{2}}\left( x,t\right) SI_{1}\left( x,t-\tau \right)$ are Lipschitz continuous functions of *S*, *E* and $I_{1}$ in the open first quadrant. In this manuscript, we assume that on ${\overline{\Omega }}$, the initial value $S_{0},E_{0},L_{0},I_{10},I_{20},R_{0}$ and $Q_{0}$ are nonnegative continuous functions, and $\int _{\Omega }I_{10}(x,s)dx>0,\int _{\Omega }I_{20}(x,s)dx>0$. Because severely infected patients $I_{2}$ are treated in the hospital and the population in compartments *Q* is cured, so we do not consider the diffusion of them in this article. Specific parameters described in Table 1.

## Results

Novel coronavirus pneumonia transmission model (1) has a disease-free equilibrium $E^{0}\left( x\right) =(S^{0}\left( x\right) ,0,0,0,0,0,0)$. In order to further study the long-term dynamic behavior of the delayed diffusive self-limiting epidemics model in temporal-spatial heterogeneous environment, we demand to prove the existence of principal eigenvalues of novel coronavirus pneumonia transmission model (1). If $\tau$ is equal to 0, linearizing the second, the third, the forth, the fifth and the sixth equations of novel coronavirus pneumonia transmission model (1) at disease-free equilibrium, we get

2 $$\begin{aligned} {\left\{ \begin{array}{ll} \frac{\partial E}{\partial t}=\nabla \cdot \left( d_{E}\left( x\right) \nabla E\right) +\beta _{1}\left( x,t\right) S^{0}E+\beta _{2}\left( x,t\right) S^{0}I_{1}+\rho _{2}\left( x,t\right) R \\ \text { \ \ \ \ \ \ }-\left[ \delta _{1}\left( x,t\right) +\gamma \left( x,t\right) +\mu \left( x,t\right) \right] E, \\ \frac{\partial L}{\partial t}=\nabla \cdot \left( d_{L}\left( x\right) \nabla L\right) +\delta _{1}\left( x,t\right) E+\delta _{2}\left( x,t\right) I_{1}-\left[ \alpha \left( x,t\right) +\theta \left( x,t\right) +\mu \left( x,t\right) \right] L, \\ \frac{\partial I_{1}}{\partial t}=\nabla \cdot \left( d_{I_{1}}\left( x\right) \nabla I_{1}\right) +\gamma \left( x,t\right) E+\theta \left( x,t\right) L+\rho _{1}\left( x,t\right) R \\ \text { \ \ \ \ \ \ }-\left[ \delta _{2}\left( x,t\right) +\omega \left( x,t\right) +\mu \left( x,t\right) +\eta _{1}\left( x,t\right) \right] I_{1}, \\ \frac{\partial I_{2}}{\partial t}=\omega \left( x,t\right) I_{1}-\left[ \phi \left( x,t\right) +\mu \left( x,t\right) +\eta _{2}\left( x,t\right) \right] I_{2}, \\ \frac{\partial R}{\partial t}=\nabla \cdot \left( d_{R}\left( x\right) \nabla R\right) +\phi \left( x,t\right) I_{2}-\left[ \rho _{1}\left( x,t\right) +\rho _{2}\left( x,t\right) +\sigma \left( x,t\right) +\mu \left( x,t\right) +\eta _{3}\left( x,t\right) \right] R, \\ \frac{\partial Q}{\partial t}=\sigma \left( x,t\right) R-\mu \left( x,t\right) Q,\text { \ \ \ \ \ \ \ }x\in \Omega ,t>0, \\ \frac{\partial S}{\partial {\mathbf {n}}}=\frac{\partial E}{\partial {\mathbf {n}}} =\frac{\partial L}{\partial {\mathbf {n}}}=\frac{\partial I_{1}}{\partial {\mathbf {n}}}=\frac{\partial I_{2}}{\partial {\mathbf {n}}}=\frac{\partial R}{ \partial {\mathbf {n}}}=\frac{\partial Q}{\partial {\mathbf {n}}}=0,\text { }x\in \partial \Omega ,t>0. \end{array}\right. } \end{aligned}$$

Let $E=e^{\uplambda t}\chi \left( x\right) ,L=e^{\uplambda t}\kappa \left( x\right) ,I_{1}=e^{\uplambda t}\varphi \left( x\right) ,I_{2}=e^{\uplambda t}\psi \left( x\right) ,R=e^{\uplambda t}\xi \left( x\right) ,Q=e^{\uplambda t}\zeta \left( x\right)$, eq. (2) can be rewritten as

3 $$\begin{aligned} {\left\{ \begin{array}{ll} \uplambda \chi \left( x\right) =\nabla \cdot \left( d_{E}\left( x\right) \nabla \chi \left( x\right) \right) +\beta _{1}\left( x,t\right) S^{0}\chi \left( x\right) +\beta _{2}\left( x,t\right) S^{0}\varphi \left( x\right) +\rho _{2}\left( x,t\right) \xi \left( x\right) \\ \text { \ \ \ \ \ \ }-\left[ \delta _{1}\left( x,t\right) +\gamma \left( x,t\right) +\mu \left( x,t\right) \right] \chi \left( x\right) , \\ \uplambda \kappa \left( x\right) =\nabla \cdot \left( d_{L}\left( x\right) \nabla \kappa \left( x\right) \right) +\delta _{1}\left( x,t\right) \chi \left( x\right) +\delta _{2}\left( x,t\right) \varphi \left( x\right) \\ \text { \ \ \ \ \ \ \ }-\left[ \alpha \left( x,t\right) +\theta \left( x,t\right) +\mu \left( x,t\right) \right] \kappa \left( x\right) , \\ \uplambda \varphi \left( x\right) =\nabla \cdot \left( d_{I_{1}}\left( x\right) \nabla \varphi \left( x\right) \right) +\gamma \left( x,t\right) \chi \left( x\right) +\theta \left( x,t\right) \kappa \left( x\right) +\rho _{1}\left( x,t\right) \xi \left( x\right) \\ \text { \ \ \ \ \ \ }-\left[ \delta _{2}\left( x,t\right) +\omega \left( x,t\right) +\mu \left( x,t\right) +\eta _{1}\left( x,t\right) \right] \varphi \left( x\right) , \\ \uplambda \psi \left( x\right) =\omega \left( x,t\right) \varphi \left( x\right) -\left[ \phi \left( x,t\right) +\mu \left( x,t\right) +\eta _{2}\left( x,t\right) \right] \psi \left( x\right) , \\ \uplambda \xi \left( x\right) =\nabla \cdot \left( d_{R}\left( x\right) \nabla \xi \left( x\right) \right) +\phi \left( x,t\right) \psi \left( x\right) \\ \text { \ \ \ \ \ \ \ }-\left[ \rho _{1}\left( x,t\right) +\rho _{2}\left( x,t\right) +\sigma \left( x,t\right) +\mu \left( x,t\right) +\eta _{3}\left( x,t\right) \right] \xi \left( x\right) , \\ \uplambda \zeta \left( x\right) =\sigma \left( x,t\right) \xi \left( x\right) -\mu \left( x,t\right) \zeta \left( x\right) ,\text { \ \ \ \ \ \ \ }x\in \Omega ,t>0, \\ \frac{\partial \chi }{\partial {\mathbf {n}}}=\frac{\partial \kappa }{\partial {\mathbf {n}}}=\frac{\partial \varphi }{\partial {\mathbf {n}}}=\frac{\partial \psi }{\partial {\mathbf {n}}}=\frac{\partial \xi }{\partial {\mathbf {n}}}=\frac{ \partial \zeta }{\partial {\mathbf {n}}}=0,\text { }x\in \partial \Omega ,t>0. \end{array}\right. } \end{aligned}$$

Denote $$\Phi \left( x\right) =\left( \chi \left( x\right) ,\kappa \left( x\right) ,\varphi \left( x\right) ,\psi \left( x\right) ,\xi \left( x\right) ,\zeta \left( x\right) \right) ^{T},$$

$$\begin{aligned} D\left( x\right) =\left[ \begin{array}{llllll} d_{E}\left( x\right) &{} 0 &{} 0 &{} 0 &{} 0 &{} 0 \\ 0 &{} d_{L}\left( x\right) &{} 0 &{} 0 &{} 0 &{} 0 \\ 0 &{} 0 &{} d_{I_{1}}\left( x\right) &{} 0 &{} 0 &{} 0 \\ 0 &{} 0 &{} 0 &{} 0 &{} 0 &{} 0 \\ 0 &{} 0 &{} 0 &{} 0 &{} d_{R}\left( x\right) &{} 0 \\ 0 &{} 0 &{} 0 &{} 0 &{} 0 &{} 0 \end{array} \right] \end{aligned}$$

and

$$\begin{aligned} M\left( x,t\right)= & {} \left( m_{ij}\left( x,t\right) \right) \\= & {} \left[ \begin{array}{llllll} m_{11}\left( x,t\right) &{} 0 &{} \beta _{2}\left( x,t\right) S^{0} &{} 0 &{} \rho _{2}\left( x,t\right) &{} 0 \\ \delta _{1}\left( x,t\right) &{} m_{22}\left( x,t\right) &{} \delta _{2}\left( x,t\right) &{} 0 &{} 0 &{} 0 \\ \gamma \left( x,t\right) &{} \theta \left( x,t\right) &{} m_{33}\left( x,t\right) &{} 0 &{} \rho _{1}\left( x,t\right) &{} 0 \\ 0 &{} 0 &{} \omega \left( x,t\right) &{} m_{44}\left( x,t\right) &{} 0 &{} 0 \\ 0 &{} 0 &{} 0 &{} \phi \left( x,t\right) &{} m_{55}\left( x,t\right) &{} 0 \\ 0 &{} 0 &{} 0 &{} 0 &{} \sigma \left( x,t\right) &{} -\mu \left( x,t\right) \end{array} \right] , \end{aligned}$$

where

$$\begin{aligned} \begin{array}{l} m_{11}\left( x,t\right) =\beta _{1}\left( x,t\right) S^{0}-\left[ \delta _{1}\left( x,t\right) +\gamma \left( x,t\right) +\mu \left( x,t\right) \right] , \\ m_{22}\left( x,t\right) =-\left[ \alpha \left( x,t\right) +\theta \left( x,t\right) +\mu \left( x,t\right) \right] , \\ m_{33}\left( x,t\right) =-\left[ \delta _{2}\left( x,t\right) +\omega \left( x,t\right) +\mu \left( x,t\right) +\eta _{1}\left( x,t\right) \right] , \\ m_{44}\left( x,t\right) =-\left[ \phi \left( x,t\right) +\mu \left( x,t\right) +\eta _{2}\left( x,t\right) \right] , \\ m_{55}\left( x,t\right) =-\left[ \rho _{1}\left( x,t\right) +\rho _{2}\left( x,t\right) +\sigma \left( x,t\right) +\mu \left( x,t\right) +\eta _{3}\left( x,t\right) \right] \end{array} \end{aligned}$$

and $m_{ij}\left( x\right) \ge 0,i\ne j,x\in {\overline{\Omega }}$. Therefore, eq. (3) can be rewritten as

4 $$\begin{aligned} {\left\{ \begin{array}{ll} \uplambda \Phi \left( x\right) =\nabla \cdot \left( D\left( x\right) \nabla \Phi \left( x\right) \right) +M\left( x,t\right) \Phi \left( x\right) ,\text { }x\in \Omega , \\ \frac{\partial \Phi }{\partial {\mathbf {n}}}=0,\text { }x\in \partial \Omega . \end{array}\right. } \end{aligned}$$

According to the Krein–Rutman theorem, we can get that eq. (4) exists a real eigenvalue $\uplambda _{*}$ and a corresponding eigenvector

$\Phi _{*}\left( x\right) =\left( \chi _{*}\left( x\right) ,\kappa _{*}\left( x\right) ,\varphi _{*}\left( x\right) ,\psi _{*}\left( x\right) ,\xi _{*}\left( x\right) ,\zeta _{*}\left( x\right) \right)$ satisfying $\Phi _{*}\left( x\right)>>0$ for all $x\in {\overline{\Omega }}$. By [16, Theorem 2.2], we can further study the principal eigenvalue of delayed system as follows:

### Lemma 1

*System (*1) *exists a principal eigenvalue *$\uplambda ^{*}$ *associated with a strictly positive eigenvector, and for any *$\tau \ge 0$, $\uplambda ^{*}$ *and *$\uplambda _{*}$ *have the same sign.*

By Lemma 1, we can get that there exists a principal eigenvalue $\uplambda ^{*}$ of system (1) and a corresponding eigenvector $\Phi ^{*}\left( x\right) =\left( \chi ^{*}\left( x\right) ,\varphi ^{*}\left( x\right) ,\varphi ^{*}\left( x\right) ,\psi ^{*}\left( x\right) ,\xi ^{*}\left( x\right) ,\zeta ^{*}\left( x\right) \right)$ satisfying $\Phi ^{*}\left( x\right)>>0$ for all $x\in {\overline{\Omega }}$ under the Neumann boundary conditions.

### Persistence of the novel coronavirus pneumonia epidemic

Here, we use the global exponential attractor theory to study the long-term dynamic behavior of the delayed reaction-diffusion self-limiting epidemic model in temporal-spatial heterogeneous environment.

Since then, we denote that $\mathbf {H=L}^{2}\left( \Omega \right)$, ${\mathbf {H}}_{1}\mathbf {=H}_{0}^{1}\left( \Omega \right) \cap {\mathbf {C}} ^{2,1}\left( \Omega \right)$,

${\mathbf {H}}^{7}=\mathbf {H\times H\times H\times H\times H\times H\times H}$ and ${\mathbf {H}}_{1}^{7}={\mathbf {H}}_{1}\mathbf {\times {\mathbf {H}}_{1}\mathbf { \times }H}_{1}\mathbf {\times H}_{1}\mathbf {\times H}_{1}\mathbf {\times H}_{1} \mathbf {\times H}_{1}$. Note that ${\mathbf {H}}^{7}$ and ${\mathbf {H}}_{1}^{7}$ are Banach spaces equipped with norm

$$\begin{aligned}&\left\| \left( S,E,L,I_{1},I_{2},Q,R\right) ^{T}\right\| _{{\mathbf {H}} ^{7}} \\&:\,=\left\| S\right\| _{{\mathbf {H}}}+\left\| E\right\| _{\mathbf {H }}+\left\| L\right\| _{{\mathbf {H}}}+\left\| I_{1}\right\| _{ {\mathbf {H}}}+\left\| I_{2}\right\| _{{\mathbf {H}}}+\left\| Q\right\| _{{\mathbf {H}}}+\left\| R\right\| _{{\mathbf {H}}} \end{aligned}$$

and

$$\begin{aligned}&\left\| \left( S,E,L,I_{1},I_{2},Q,R\right) ^{T}\right\| _{{\mathbf {H}} _{1}^{7}} \\&:=\left\| S\right\| _{{\mathbf {H}}_{1}}+\left\| E\right\| _{ {\mathbf {H}}_{1}}+\left\| L\right\| _{{\mathbf {H}}_{1}}+\left\| I_{1}\right\| _{{\mathbf {H}}_{1}}+\left\| I_{2}\right\| _{{\mathbf {H}} _{1}}+\left\| R\right\| _{{\mathbf {H}}_{1}}+\left\| Q\right\| _{ {\mathbf {H}}_{1}}. \end{aligned}$$

For any given continuous function *f* on ${\overline{\Omega }}\times \left( 0,+\infty \right)$, we denote

$$\begin{aligned} f^{*}=\underset{x\in {\overline{\Omega }},t>0}{\sup }f\left( x,t\right) \text { and }f_{*}=\underset{x\in {\overline{\Omega }},t>0}{\inf }f\left( x,t\right) . \end{aligned}$$

For the spatial heterogeneous diffusion coefficients, we also denote that

$$\begin{aligned} \left( d_{S}\right) _{*}= & {} \underset{x\in {\overline{\Omega }}}{\inf } d_{S}\left( x\right) ,\left( d_{E}\right) _{*}=\underset{x\in \overline{ \Omega }}{\inf }d_{E}\left( x\right) ,\left( d_{L}\right) _{*}=\underset{ x\in {\overline{\Omega }}}{\inf }d_{L}\left( x\right) , \\ \left( d_{I_{1}}\right) _{*}= & {} \underset{x\in {\overline{\Omega }}}{\inf } d_{I_{1}}\left( x\right) ,\left( d_{R}\right) _{*}=\underset{x\in {\overline{\Omega }}}{\inf }d_{R}\left( x\right) . \end{aligned}$$

Next, we first investigate the existence, positivity and boundedness of the global solution of the novel coronavirus pneumonia transmission model (1).

#### Theorem 2

*For each* $(S_{0}(x),E_{0}(x),L_{0}(x),I_{10}(x),I_{20}(x),R_{0}\left( x\right) ,Q_{0}(x))\in {\mathbf {C}}({\overline{\Omega }}\times \left[ -\tau ,0\right] )$*, novel coronavirus pneumonia system (*1*) exists a positive and bounded global solution* $\left( S(x,t),E(x,t),L(x,t),I_{1}(x,t),I_{2}(x,t),R\left( x,t\right) ,Q(x,t)\right) \in {\mathbf {C}}^{2,1}(\Omega \times \left( -\tau ,\infty \right) )$.

#### Proof

Since

$$\begin{aligned} {\mathcal {L}}=\left( \nabla \cdot \left( d_{S}\left( x\right) \nabla \right) ,\nabla \cdot \left( d_{E}\left( x\right) \nabla \right) ,\nabla \cdot \left( d_{L}\left( x\right) \nabla \right) ,\nabla \cdot \left( d_{I_{1}}\left( x\right) \nabla \right) ,0,\nabla \cdot \left( d_{R}\left( x\right) \nabla \right) ,0\right) \end{aligned}$$

is a symmetrical sectorial operator and all eigenvalues of $\mathcal {L}$ are

$$\begin{aligned} 0>\uplambda _{1}\ge \uplambda _{2}\ge ...\ge \uplambda _{k}>...,\text { }\uplambda _{k}\rightarrow -\infty \text { }\left( k\rightarrow \infty \right) , \\ \begin{array}{l} G\left( S,E,L,I_{1},I_{2},R,Q\right) :=\left( g_{1}\left( S,E,L,I_{1},I_{2},R,Q\right) ,g\left( S,E,L,I_{1},I_{2},R,Q\right) ,\right. \\ g_{3}\left( S,E,L,I_{1},I_{2},R,Q\right) ,g_{4}\left( S,E,L,I_{1},I_{2},R,Q\right) ,g_{5}\left( S,E,L,I_{1},I_{2},R,Q\right) \\ \left. g_{6}\left( S,E,L,I_{1},I_{2},R,Q\right) ,g_{7}\left( S,E,L,I_{1},I_{2},R,Q\right) \right) ^{T}, \end{array} \end{aligned}$$

where

$$\begin{aligned} \begin{array}{l} g_{1}\left( S,E,L,I_{1},I_{2},R,Q\right) =\Lambda \left( x,t\right) -\beta _{1}\left( x,t\right) SE-\beta _{2}\left( x,t\right) SI_{1}\left( x,t-\tau \right) \\ \text { \ \ \ \ \ \ \ \ \ \ \ \ \ \ \ \ \ \ \ \ \ \ \ \ \ \ \ \ \ \ \ \ }+\alpha \left( x,t\right) L-\mu \left( x,t\right) S, \\ g_{2}\left( S,E,L,I_{1},I_{2},R,Q\right) =\beta _{1}\left( x,t\right) SE+\beta _{2}\left( x,t\right) SI_{1}\left( x,t-\tau \right) +\rho _{2}\left( x,t\right) R \\ \text { \ \ \ \ \ \ \ \ \ \ \ \ \ \ \ \ \ \ \ \ \ \ \ \ \ \ \ \ \ \ \ \ }-\left[ \delta _{1}\left( x,t\right) +\gamma \left( x,t\right) +\mu \left( x,t\right) \right] E, \\ g_{3}\left( S,E,L,I_{1},I_{2},R,Q\right) =\delta _{1}\left( x,t\right) E+\delta _{2}\left( x,t\right) I_{1}-\left[ \alpha \left( x,t\right) +\theta \left( x,t\right) +\mu \left( x,t\right) \right] L, \\ g_{4}\left( S,E,L,I_{1},I_{2},R,Q\right) =\gamma \left( x,t\right) E+\theta \left( x,t\right) L+\rho _{1}\left( x,t\right) R \\ \text { \ \ \ \ \ \ \ \ \ \ \ \ \ \ \ \ \ \ \ \ \ \ \ \ \ \ \ \ \ \ \ \ }-\left[ \delta _{2}\left( x,t\right) +\omega \left( x,t\right) +\mu \left( x,t\right) +\eta _{1}\left( x,t\right) \right] I_{1}, \\ g_{5}\left( S,E,L,I_{1},I_{2},R,Q\right) =\omega \left( x,t\right) I_{1}- \left[ \phi \left( x,t\right) +\mu \left( x,t\right) +\eta _{2}\left( x,t\right) \right] I_{2}, \\ g_{6}\left( S,E,L,I_{1},I_{2},R,Q\right) =\phi \left( x,t\right) I_{2}-\left[ \rho _{1}\left( x,t\right) +\rho _{2}\left( x,t\right) +\sigma \left( x,t\right) \right. \\ \text { \ \ \ \ \ \ \ \ \ \ \ \ \ \ \ \ \ \ \ \ \ \ \ \ \ \ \ \ \ \ \ \ }\left. +\mu \left( x,t\right) +\eta _{3}\left( x,t\right) \right] R, \\ g_{7}\left( S,E,L,I_{1},I_{2},R,Q\right) =\sigma \left( x,t\right) R-\mu \left( x,t\right) Q \end{array} \end{aligned}$$

be quasimonotone and satisfy the locally Lipschitz conditions, then by [17, Theorem 11.3.5] and [20, Theorem 2.3], we can deduce that novel coronavirus pneumonia transmission model (1) exists a global solution

$\left( S(x,t),E(x,t),L(x,t),I_{1}(x,t),I_{2}(x,t),R\left( x,t\right) ,Q(x,t)\right) \in {\mathbf {C}}^{2,1}(\Omega \times \left( 0,\infty \right) )$. The same as the method in [21, Lemma 2.1 and Theorem 2.2] , we can prove that the global solution of the novel coronavirus pneumonia transmission model (1) is positive. Next, we consider the following total population at time *t*. Define

$$\begin{aligned} U\left( t\right) =\int _{\Omega }\left[ S(x,t)+E(x,t)+L(x,t)+I_{1}(x,t)+I_{2}(x,t)+R\left( x,t\right) +Q(x,t)\right] dx. \end{aligned}$$

Take the derivative of $U\left( t\right)$ to get

$$\begin{aligned} \frac{dU\left( t\right) }{dt}=&\int _{\Omega }\left[ \frac{\partial }{ \partial t}S\left( x,t\right) +\frac{\partial }{\partial t}E\left( x,t\right) +\frac{\partial }{\partial t}L\left( x,t\right) +\frac{\partial }{ \partial t}I_{1}\left( x,t\right) +\frac{\partial }{\partial t}I_{2}\left( x,t\right) \right. \\&\left. +\frac{\partial }{\partial t}R\left( x,t\right) +\frac{\partial }{ \partial t}Q\left( x,t\right) \right] dx \\ =&\int _{\Omega }\left\{ \nabla \cdot \left( d_{S}\left( x\right) \nabla S\right) +\nabla \cdot \left( d_{E}\left( x\right) \nabla E\right) +\nabla \cdot \left( d_{L}\left( x\right) \nabla L\right) \right. \\&+\nabla \cdot \left( d_{I_{1}}\left( x\right) \nabla I_{1}\right) +\nabla \cdot \left( d_{R}\left( x\right) \nabla R\right) \\&+\Lambda \left( x,t\right) -\mu \left( x,t\right) S-\mu \left( x,t\right) E-\mu \left( x,t\right) L-\left[ \mu \left( x\right) +\eta _{1}\left( x\right) \right] I_{1} \\&\left. -\left[ \mu \left( x\right) +\eta _{2}\left( x\right) \right] I_{2}- \left[ \mu \left( x\right) +\eta _{2}\left( x\right) \right] R-\mu \left( x\right) Q\right\} dx \\ \le&\int _{\Omega }\left\{ \nabla \cdot \left( d_{S}\left( x\right) \nabla S\right) +\nabla \cdot \left( d_{E}\left( x\right) \nabla E\right) +\nabla \cdot \left( d_{L}\left( x\right) \nabla L\right) \right. \\&+\nabla \cdot \left( d_{I_{1}}\left( x\right) \nabla I_{1}\right) +\nabla \cdot \left( d_{R}\left( x\right) \nabla R\right) \\&+\int _{\Omega }\left\{ \Lambda ^{*}-\mu _{*}\left[ S+E+L+I_{1}+I_{2}+R+Q\right] \right\} dx \\ \le&\Lambda ^{*}\left| \Omega \right| -\mu _{*}U\left( t\right) . \end{aligned}$$

According to the Gronwall’s inequality in differential form [21, Lemma 2.2], we can obtain that

$$\begin{aligned} U\left( t\right) \le U\left( 0\right) e^{-\mu _{*}t}+\frac{\Lambda ^{*}\left| \Omega \right| }{\mu _{*}}\left( 1-e^{-\mu _{*}t}\right) . \end{aligned}$$

So $$U(t)\le \max \left\{ U(0),\frac{\Lambda ^{*}\left| \Omega \right| }{\mu _{*}}\right\}$$, where

$$\begin{aligned}&U(0) \\ =&\int _{\Omega }\left[ S\left( x,0\right) +E\left( x,0\right) +L\left( x,0\right) +I_{1}\left( x,0\right) +I_{2}\left( x,0\right) +R\left( x,0\right) +Q\left( x,0\right) \right] dx \\ \le&\int _{\Omega }\left\| S\left( x,0\right) +E\left( x,0\right) +L\left( x,0\right) +I_{1}\left( x,0\right) +I_{2}\left( x,0\right) +R\left( x,0\right) +Q\left( x,0\right) \right\| _{L^{\infty }(\Omega )}dx \\ =&\left\| S\left( x,0\right) +E\left( x,0\right) +L\left( x,0\right) +I_{1}\left( x,0\right) +I_{2}\left( x,0\right) +R\left( x,0\right) +Q\left( x,0\right) \right\| _{L^{\infty }(\Omega )}\left| \Omega \right| . \end{aligned}$$

Hence, $U(t)=\int _{\Omega }\left( S+E+L+I_{1}+I_{2}+R+Q\right) dx$ is bounded. By the positivity of the solution of the novel coronavirus pneumonia transmission model (1), we obtain that

$$\begin{aligned}&\left\| S+E+L+I_{1}+I_{2}+R+Q\right\| _{L^{1}(\Omega )} \\ =&\int _{\Omega }\left| S+E+L+I_{1}+I_{2}+R+Q\right| \left( x,t\right) dx \\ =&\int _{\Omega }\left( S+E+L+I_{1}+I_{2}+R+Q\right) \left( x,t\right) dx \\ \le&\max \left\{ \left\| \begin{array}{l} S\left( x,0\right) +E\left( x,0\right) +L\left( x,0\right) \\ +I_{1}\left( x,0\right) +I_{2}\left( x,0\right) +R\left( x,0\right) +Q\left( x,0\right) \end{array} \right\| _{L^{\infty }(\Omega )}\left| \Omega \right| ,\frac{ \Lambda ^{*}\left| \Omega \right| }{\mu _{*}}\right\} . \end{aligned}$$

We denote that $$K=\max \left\{ \left\| \begin{array}{l} S\left( x,0\right) +E\left( x,0\right) +L\left( x,0\right) \\ +I_{1}\left( x,0\right) +I_{2}\left( x,0\right) +R\left( x,0\right) +Q\left( x,0\right) \end{array} \right\| _{L^{\infty }(\Omega )}\left| \Omega \right| ,\frac{ \Lambda ^{*}\left| \Omega \right| }{\mu _{*}}\right\}$$, then we know

$$\begin{aligned} \int _{\Omega }(S+E+L+I_{1}+I_{2}+R+Q)dx\le K. \end{aligned}$$

Due to [11, Theorem 1 and Corollary 1], there exists a positive constant $K^{*}$ depending on *K* such that

$$\begin{aligned} \left\| S+E+L+I_{1}+I_{2}+R+Q\right\| _{L^{\infty }(\Omega )}\le K^{*}. \end{aligned}$$

Thus, $S(x,t),E(x,t),L(x,t),I_{1}(x,t),I_{2}(x,t),R\left( x,t\right) ,Q(x,t)$ are uniformly bounded on ${\overline{\Omega }}$. Hence, the global solution of novel coronavirus pneumonia transmission model (1) is positive and uniformly bounded. $\square$

#### Theorem 3

*There exists a global exponential attractor* ${\mathcal {A}} ^{*}$ *of novel coronavirus pneumonia transmission model (*1),* it exponential attracts any bounded set in* ${\mathbf {H}}^{7}$.

#### *Proof*

For the novel coronavirus pneumonia diffusion system (1), we first confirm the [22, condition (2.3)]. Since

$$\begin{aligned}&\left\langle \begin{array}{cc} \begin{array}{l} \nabla \cdot \left( d_{S}\left( x\right) \nabla S\right) +\Lambda \left( x,t\right) -\beta _{1}\left( x,t\right) SE-\beta _{2}\left( x,t\right) SI_{1}\left( x,t-\tau \right) \\ +\alpha \left( x,t\right) L-\mu \left( x,t\right) S \end{array} ,&S \end{array} \right\rangle _{{\mathbf {H}}} \\ =&\int _{\Omega }\nabla \cdot \left( d_{S}\left( x\right) \nabla S\right) \cdot Sdx+\int _{\Omega }\Lambda \left( x,t\right) Sdx-\int _{\Omega }\beta _{1}\left( x,t\right) S^{2}Edx \\&-\int _{\Omega }\beta _{2}\left( x,t\right) S^{2}I_{1}\left( x,t-\tau \right) dx+\int _{\Omega }\alpha \left( x,t\right) SLdx-\int _{\Omega }\mu \left( x,t\right) S^{2}dx \\ =&\int _{\Omega }\sum _{i=1}^{n}S\cdot \frac{\partial }{\partial x_{i}}\left( d_{S}\left( x\right) \frac{\partial S}{\partial x_{i}}\right) dx+\int _{\Omega }\Lambda \left( x,t\right) Sdx-\int _{\Omega }\beta _{1}\left( x,t\right) S^{2}Edx \\&-\int _{\Omega }\beta _{2}\left( x,t\right) S^{2}I_{1}\left( x,t-\tau \right) dx+\int _{\Omega }\alpha \left( x,t\right) SLdx-\int _{\Omega }\mu \left( x,t\right) S^{2}dx \\ =&\sum _{i=1}^{n}\int _{\Omega }S\cdot \frac{\partial }{\partial x_{i}}\left( d_{S}\left( x\right) \frac{\partial S}{\partial x_{i}}\right) dx+\int _{\Omega }\Lambda \left( x,t\right) Sdx-\int _{\Omega }\beta _{1}\left( x,t\right) S^{2}Edx \\&-\int _{\Omega }\beta _{2}\left( x,t\right) S^{2}I_{1}\left( x,t-\tau \right) dx+\int _{\Omega }\alpha \left( x,t\right) SLdx-\int _{\Omega }\mu \left( x,t\right) S^{2}dx \\ =&-\sum _{i=1}^{n}\int _{\Omega }d_{S}\left( x\right) \left( \frac{\partial S }{\partial x_{i}}\right) ^{2}dx+\sum _{i=1}^{n}\int _{\partial \Omega }S\cdot \left( d_{S}\left( x,t\right) \frac{\partial S}{\partial x_{i}}\right) \cdot {\mathbf {n}}_{x_{i}}d{\mathbf {s}}+\int _{\Omega }\Lambda \left( x,t\right) Sdx \\&-\int _{\Omega }\beta _{1}\left( x,t\right) S^{2}Edx-\int _{\Omega }\beta _{2}\left( x,t\right) S^{2}I_{1}\left( x,t-\tau \right) dx+\int _{\Omega }\alpha \left( x,t\right) SLdx-\int _{\Omega }\mu \left( x,t\right) S^{2}dx \\ =&-\int _{\Omega }d_{S}\left( x\right) \sum _{i=1}^{n}\left( \frac{\partial S }{\partial x_{i}}\right) ^{2}dx+\int _{\partial \Omega }Sd_{S}\left( x,t\right) \frac{\partial S}{\partial {\mathbf {n}}}d{\mathbf {s}}+\int _{\Omega }\Lambda \left( x,t\right) Sdx \\&-\int _{\Omega }\beta _{1}\left( x,t\right) S^{2}Edx-\int _{\Omega }\beta _{2}\left( x,t\right) S^{2}I_{1}\left( x,t-\tau \right) dx \\&+\int _{\Omega }\alpha \left( x,t\right) SLdx-\int _{\Omega }\mu \left( x,t\right) S^{2}dx \\ =&-\int _{\Omega }d_{S}\left( x\right) \left| \nabla S\right| ^{2}dx+\int _{\Omega }\Lambda \left( x,t\right) Sdx-\int _{\Omega }\beta _{1}\left( x,t\right) S^{2}Edx \\&-\int _{\Omega }\beta _{2}\left( x,t\right) S^{2}I_{1}\left( x,t-\tau \right) dx+\int _{\Omega }\alpha \left( x,t\right) SLdx-\int _{\Omega }\mu \left( x,t\right) S^{2}dx \\ \le&-\left( d_{S}\right) _{*}\int _{\Omega }\left| \nabla S\right| ^{2}dx+\Lambda ^{*}\int _{\Omega }Sdx+\alpha ^{*}\int _{\Omega }SLdx \\ =&-\left( d_{S}\right) _{*}\left\| S\right\| _{{\mathbf {H}}_{\frac{1 }{2}}}^{2}+\Lambda ^{*}\int _{\Omega }Sdx+\alpha ^{*}\int _{\Omega }SLdx, \end{aligned}$$

$$\begin{aligned}&\left\langle \begin{array}{ll} \begin{array}{l} \nabla \cdot \left( d_{E}\left( x\right) \nabla E\right) +\beta _{1}\left( x,t\right) SE+\beta _{2}\left( x,t\right) SI_{1}\left( x,t-\tau \right) +\rho _{2}\left( x,t\right) R \\ -\left[ \delta _{1}\left( x,t\right) +\gamma \left( x,t\right) +\mu \left( x,t\right) \right] E \end{array} ,&E \end{array} \right\rangle _{{\mathbf {H}}} \\ =&\int _{\Omega }\nabla \cdot \left( d_{E}\left( x\right) \nabla E\right) \cdot Edx+\int _{\Omega }\beta _{1}\left( x,t\right) SE^{2}dx+\int _{\Omega }\beta _{2}\left( x,t\right) SEI_{1}\left( x,t-\tau \right) dx \\&+\int _{\Omega }\rho _{2}\left( x,t\right) ERdx-\int _{\Omega }\left[ \delta _{1}\left( x,t\right) +\gamma \left( x,t\right) +\mu \left( x,t\right) \right] E^{2}dx \\ \le&-\left( d_{E}\right) _{*}\left\| E\right\| _{{\mathbf {H}}_{ \frac{1}{2}}}^{2}+\int _{\Omega }\beta _{1}\left( x,t\right) SE^{2}dx+\int _{\Omega }\beta _{2}\left( x,t\right) SEI_{1}\left( x,t-\tau \right) dx \\&+\int _{\Omega }\rho _{2}\left( x,t\right) ERdx \\ \le&-\left( d_{E}\right) _{*}\left\| E\right\| _{{\mathbf {H}}_{ \frac{1}{2}}}^{2}+\beta _{1}^{*}\int _{\Omega }SE^{2}dx+\beta _{2}^{*}\int _{\Omega }SEI_{1}\left( x,t-\tau \right) dx+\rho _{2}^{*}\int _{\Omega }ERdx, \end{aligned}$$

$$\begin{aligned}&\left\langle \begin{array}{ll} \begin{array}{l} \nabla \cdot \left( d_{L}\left( x\right) \nabla L\right) +\delta _{1}\left( x,t\right) E+\delta _{2}\left( x,t\right) I_{1} \\ -\left[ \alpha \left( x,t\right) +\theta \left( x,t\right) +\mu \left( x,t\right) \right] L \end{array} ,&L \end{array} \right\rangle _{{\mathbf {H}}} \\ =&\int _{\Omega }\nabla \cdot \left( d_{L}\left( x\right) \nabla L\right) \cdot Ldx+\int _{\Omega }\delta _{1}\left( x,t\right) ELdx+\int _{\Omega }\delta _{2}\left( x,t\right) LI_{1}dx \\&-\int _{\Omega }\left[ \alpha \left( x,t\right) +\theta \left( x,t\right) +\mu \left( x,t\right) \right] L^{2}dx \\ \le&-\left( d_{L}\right) _{*}\left\| L\right\| _{{\mathbf {H}}_{ \frac{1}{2}}}^{2}+\delta _{1}^{*}\int _{\Omega }ELdx+\delta _{2}^{*}\int _{\Omega }LI_{1}dx, \\&\left\langle \begin{array}{ll} \begin{array}{l} \nabla \cdot \left( d_{I_{1}}\left( x\right) \nabla I_{1}\right) +\gamma \left( x,t\right) E+\theta \left( x,t\right) L+\rho _{1}\left( x,t\right) R \\ -\left[ \delta _{2}\left( x,t\right) +\omega \left( x,t\right) +\mu \left( x,t\right) +\eta _{1}\left( x,t\right) \right] I_{1} \end{array} ,&I_{1} \end{array} \right\rangle _{{\mathbf {H}}} \\ =&\int _{\Omega }\nabla \cdot \left( d_{I_{1}}\left( x\right) \nabla I_{1}\right) \cdot I_{1}dx+\int _{\Omega }\gamma \left( x,t\right) EI_{1}dx+\int _{\Omega }\theta \left( x,t\right) LI_{1}dx \\&+\int _{\Omega }\rho _{1}\left( x,t\right) I_{1}Rdx-\int _{\Omega }\left[ \delta _{2}\left( x,t\right) +\omega \left( x,t\right) +\mu \left( x,t\right) +\eta _{1}\left( x,t\right) \right] I_{1}^{2}dx \\ \le&-\left( d_{I_{1}}\right) _{*}\left\| I_{1}\right\| _{ {\mathbf {H}}_{\frac{1}{2}}}^{2}+\gamma ^{*}\int _{\Omega }EI_{1}dx+\theta ^{*}\int _{\Omega }LI_{1}dx+\rho _{1}^{*}\int _{\Omega }I_{1}Rdx, \end{aligned}$$

$$\begin{aligned}&\left\langle \omega \left( x,t\right) I_{1}-\left[ \phi \left( x,t\right) +\mu \left( x,t\right) +\eta _{2}\left( x,t\right) \right] I_{2},I_{2}\right\rangle _{{\mathbf {H}}} \\ =&\int _{\Omega }\omega \left( x,t\right) I_{1}I_{2}dx-\int _{\Omega }\left[ \phi \left( x,t\right) +\mu \left( x,t\right) +\eta _{2}\left( x,t\right) \right] I_{2}^{2}dx \\ \le&\omega ^{*}\int _{\Omega }I_{1}I_{2}dx, \\&\left\langle \begin{array}{ll} \begin{array}{l} \nabla \cdot \left( d_{R}\left( x\right) \nabla R\right) +\phi \left( x,t\right) I_{2}-\left[ \rho _{1}\left( x,t\right) +\rho _{2}\left( x,t\right) +\sigma \left( x,t\right) \right. \\ \left. +\mu \left( x,t\right) +\eta _{3}\left( x,t\right) \right] R \end{array} ,&R \end{array} \right\rangle _{{\mathbf {H}}} \\ =&\int _{\Omega }\nabla \cdot \left( d_{R}\left( x\right) \nabla R\right) \cdot Rdx+\int _{\Omega }\phi \left( x,t\right) I_{2}Rdx \\&-\int _{\Omega }\left[ \rho _{1}\left( x,t\right) +\rho _{2}\left( x,t\right) +\sigma \left( x,t\right) +\mu \left( x,t\right) +\eta _{3}\left( x,t\right) \right] R^{2}dx \\ \le&-\left( d_{R}\right) _{*}\left\| R\right\| _{{\mathbf {H}}_{ \frac{1}{2}}}^{2}+\phi ^{*}\int _{\Omega }I_{2}Rdx, \end{aligned}$$

$$\begin{aligned}&\left\langle \sigma \left( x,t\right) R-\mu \left( x,t\right) Q,Q\right\rangle _{{\mathbf {H}}} \\ =&\int _{\Omega }\sigma \left( x,t\right) RQdx-\int _{\Omega }\mu \left( x,t\right) Q^{2}dx \\ \le&\sigma ^{*}\int _{\Omega }RQdx. \end{aligned}$$

In view of Theorem 2, we know $\left( S(x,t),I_{1}(x,t),I_{2}(x,t),R\left( x,t\right) ,Q(x,t)\right)$ is uniformly bounded, hence, [22, condition (2.3)] holds. Moreover, denote that

$u=\left( S_{1},E_{1},L_{1},I_{11},I_{21},R_{1},Q_{1}\right) ,v=\left( S_{2},E_{2},L_{2},I_{12},I_{22},R_{2},Q_{2}\right)$, we can verify that there is a constant ${\widehat{L}}$, such that

$$\begin{aligned} \begin{array}{ll} &{} \left\| G\left( t,u\right) -G\left( t,v\right) \right\| _{{\mathbf {H}} ^{7}} \\ = &{} \left\| \left( g_{1}\left( t,u\right) -g_{1}\left( t,v\right) \right) ,\left( g_{2}\left( t,u\right) -g_{2}\left( t,v\right) \right) ,\left( g_{3}\left( t,u\right) -g_{3}\left( t,v\right) \right) ,\left( g_{4}\left( t,u\right) -g_{4}\left( t,v\right) \right) ,\right. \\ &{} \left. \left( g_{5}\left( t,u\right) -g_{5}\left( t,v\right) \right) ,\left( g_{6}\left( t,u\right) -g_{6}\left( t,v\right) \right) ,\left( g_{7}\left( t,u\right) -g_{7}\left( t,v\right) \right) \right\| _{\mathbf { H}^{7}} \\ = &{} \left\| g_{1}\left( t,u\right) -g_{1}\left( t,v\right) \right\| _{ {\mathbf {H}}}+\left\| g_{2}\left( t,u\right) -g_{2}\left( t,v\right) \right\| _{{\mathbf {H}}}+\left\| g_{3}\left( t,u\right) -g_{3}\left( t,v\right) \right\| _{{\mathbf {H}}} \\ &{} +\left\| g_{4}\left( t,u\right) -g_{4}\left( t,v\right) \right\| _{ {\mathbf {H}}}+\left\| g_{5}\left( t,u\right) -g_{5}\left( t,v\right) \right\| _{{\mathbf {H}}}+\left\| g_{6}\left( t,u\right) -g_{6}\left( t,v\right) \right\| _{{\mathbf {H}}} \\ &{} +\left\| g_{7}\left( t,u\right) -g_{7}\left( t,v\right) \right\| _{ {\mathbf {H}}} \\ \le &{} {\widehat{L}}\cdot \left\| u-v\right\| _{{\mathbf {H}}^{7}}. \end{array} \end{aligned}$$

Hence, Lipschitz condition is well verified. Since that ${\mathcal {L}}=\left( \nabla \cdot \left( d_{S}\left( x\right) \nabla \right) ,\nabla \cdot \left( d_{E}\left( x\right) \nabla \right) ,\nabla \cdot \left( d_{L}\left( x\right) \nabla \right) ,\nabla \cdot \left( d_{I_{1}}\left( x\right) \nabla \right) ,0,\nabla \cdot \left( d_{R}\left( x\right) \nabla \right) ,0\right)$ is a symmetrical sectorial operator and all eigenvalues of ${\mathcal {L}}$ are

$$\begin{aligned} 0>\uplambda _{1}\ge \uplambda _{2}\ge ...\ge \uplambda _{k}>...,\uplambda _{k}\rightarrow -\infty \text { }\left( k\rightarrow \infty \right) , \end{aligned}$$

therefore, by [22, Lemma 2.5], the novel coronavirus pneumonia transmission model (1) has a invariant set, it exponential attracts any bounded set in ${\mathbf {H}}^{7}$. From [22, Theorem 2.7], we can gain that the novel coronavirus pneumonia transmission model (1) has a global exponential attractor $\mathcal{A}^*$and $\dim _{F}\left( {\mathcal {A}}^{*}\right) =d_{0}<\infty$. $\square$

After getting the global exponential attractor, we can discuss the stability and persists uniformly of the novel coronavirus pneumonia.

#### Theorem 4

1. If $$\uplambda ^{*}<0$$*, then*
  $$\begin{aligned} \underset{t\rightarrow \infty }{\lim }S\left( x,t\right)= & {} S^{0}\left( x\right) ,\underset{t\rightarrow \infty }{\lim }E\left( x,t\right) =0, \underset{t\rightarrow \infty }{\lim }L\left( x,t\right) =0,\underset{ t\rightarrow \infty }{\lim }I_{1}\left( x,t\right) =0, \\ \underset{t\rightarrow \infty }{\lim }I_{2}\left( x,t\right)= & {} 0,\underset{ t\rightarrow \infty }{\lim }R\left( x,t\right) =0,\underset{t\rightarrow \infty }{\lim }Q\left( x,t\right) =0 \end{aligned}$$
  in ${\mathbf {H}}$, *that is the COVID-19 epidemic will be effectively controlled and eventually eliminated*.
2. If $\uplambda ^{*}>0$, *then there exists a positive function* $\varrho \left( x\right)$ *independent of the initial data, such that any solution* $(S,E,L,I_{1},I_{2},R,Q)$ satisfies
  $$\begin{aligned} \underset{t\rightarrow \infty }{\lim \inf }S\left( x,t\right)\ge & {} \varrho \left( x\right) ,\underset{t\rightarrow \infty }{\lim \inf }E\left( x,t\right) \ge \varrho \left( x\right) ,\underset{t\rightarrow \infty }{ \lim \inf }L\left( x,t\right) \ge \varrho \left( x\right) , \\ \underset{t\rightarrow \infty }{\lim \inf }I_{1}\left( x,t\right)\ge & {} \varrho \left( x\right) ,\underset{t\rightarrow \infty }{\lim \inf } I_{2}\left( x,t\right) \ge \varrho \left( x\right) , \\ \underset{t\rightarrow \infty }{\lim \inf }R\left( x,t\right)\ge & {} \varrho \left( x\right) ,\underset{t\rightarrow \infty }{\lim \inf }Q\left( x,t\right) \ge \varrho \left( x\right) \end{aligned}$$
  for $x\in {\overline{\Omega }}$, *that is the COVID-19 epidemic will persists uniformly*.

#### *Proof*

1. Suppose $$\uplambda ^{*}<0$$. We intend to use the comparison principle to prove that $$E\left( x,t\right) \rightarrow 0,L\left( x,t\right) \rightarrow 0,I_{1}\left( x,t\right) \rightarrow 0,I_{2}\left( x,t\right) \rightarrow 0,Q\left( x,t\right) \rightarrow 0,R\left( x,t\right) \rightarrow 0$$ as $$t\rightarrow \infty$$ for each $$x\in \Omega$$. First, we observe from the system (1) that
  $$\begin{aligned} {\left\{ \begin{array}{ll} \frac{\partial E}{\partial t}\le \nabla \cdot \left( d_{E}\left( x\right) \nabla E\right) +\left\{ \beta _{1}\left( x,t\right) K^{*}-\left[ \delta _{1}\left( x,t\right) +\gamma \left( x,t\right) +\mu \left( x,t\right) \right] \right\} E \\ \text { \ \ \ \ \ \ }+\beta _{2}\left( x,t\right) K^{*}I_{1}+\rho _{2}\left( x,t\right) R, \\ \frac{\partial L}{\partial t}\le \nabla \cdot \left( d_{L}\left( x\right) \nabla L\right) +\delta _{1}\left( x,t\right) E+\delta _{2}\left( x,t\right) I_{1} \\ \text { \ \ \ \ \ \ }-\left[ \alpha \left( x,t\right) +\theta \left( x,t\right) +\mu \left( x,t\right) \right] L, \\ \frac{\partial I_{1}}{\partial t}\le \nabla \cdot \left( d_{I_{1}}\left( x\right) \nabla I_{1}\right) +\gamma \left( x,t\right) E+\theta \left( x,t\right) L+\rho _{1}\left( x,t\right) R \\ \text { \ \ \ \ \ \ }-\left[ \delta _{2}\left( x,t\right) +\omega \left( x,t\right) +\mu \left( x,t\right) +\eta _{1}\left( x,t\right) \right] I_{1}, \\ \frac{\partial I_{2}}{\partial t}\le \omega \left( x,t\right) I_{1}-\left[ \phi \left( x,t\right) +\mu \left( x,t\right) +\eta _{2}\left( x,t\right) \right] I_{2}, \\ \frac{\partial R}{\partial t}\le \nabla \cdot \left( d_{R}\left( x\right) \nabla R\right) +\phi \left( x,t\right) I_{2} \\ \text { \ \ \ \ \ \ }-\left[ \rho _{1}\left( x,t\right) +\rho _{2}\left( x,t\right) +\sigma \left( x,t\right) +\mu \left( x,t\right) +\eta _{3}\left( x,t\right) \right] R, \\ \frac{\partial Q}{\partial t}\le \sigma \left( x,t\right) R-\mu \left( x,t\right) Q. \end{array}\right. } \end{aligned}$$
  Next, let us define $$\left( {\widetilde{E}}\left( x,t\right) ,{\widetilde{L}} \left( x,t\right) ,\widetilde{I_{1}}\left( x,t\right) ,\widetilde{I_{2}} \left( x,t\right) ,{\widetilde{R}}\left( x,t\right) ,{\widetilde{Q}}\left( x,t\right) \right) = \left( Me^{\uplambda ^{*}t}\chi ^{*}\left( x\right) ,Me^{\uplambda ^{*}t}\kappa ^{*}\left( x\right) ,Me^{\uplambda ^{*}t}\varphi ^{*}\left( x\right) ,Me^{\uplambda ^{*}t}\psi ^{*}\left( x\right) ,Me^{\uplambda ^{*}t}\xi ^{*}\left( x\right) ,Me^{\uplambda ^{*}t}\zeta ^{*}\left( x\right) \right)$$ where $$\uplambda ^{*}<0,\chi ^{*}\left( x\right)>>0,\kappa ^{*}\left( x\right)>>0,\varphi ^{*}\left( x\right)>>0,\psi ^{*}\left( x\right)>>0,\xi ^{*}\left( x\right)>>0,\zeta ^{*}\left( x\right)>>0$$ are the eigenvalue and eigenvectors in eq. (3) and *M* is chosen so large that $$E\left( x,0\right) \le {\widetilde{E}}\left( x,0\right) ,L\left( x,0\right) \le {\widetilde{L}}\left( x,0\right) ,I_{1}\left( x,0\right) \le \widetilde{I_{1}}\left( x,0\right) ,I_{2}\left( x,0\right) \le \widetilde{I_{2}}\left( x,0\right) ,R\left( x,0\right) \le \widetilde{R }\left( x,0\right) ,Q\left( x,0\right) \le {\widetilde{Q}}\left( x,0\right)$$ for every $$x\in \Omega$$. It can be shown that $$\left( {\widetilde{E}}\left( x,t\right) ,{\widetilde{L}}\left( x,t\right) ,\widetilde{I_{1}}\left( x,t\right) ,\widetilde{I_{2}}\left( x,t\right) ,{\widetilde{R}}\left( x,t\right) ,{\widetilde{Q}}\left( x,t\right) \right)$$ satisfies
  $$\begin{aligned} {\left\{ \begin{array}{ll} \frac{\partial {\widetilde{E}}}{\partial t}=\nabla \cdot \left( d_{E}\left( x\right) \nabla {\widetilde{E}}\right) +\left\{ \beta _{1}\left( x,t\right) K^{*}-\left[ \delta _{1}\left( x,t\right) +\gamma \left( x,t\right) +\mu \left( x,t\right) \right] \right\} {\widetilde{E}} \\ \text { \ \ \ \ \ \ }+\beta _{2}\left( x,t\right) K^{*}\widetilde{I_{1}} +\rho _{2}\left( x,t\right) {\widetilde{R}}, \\ \frac{\partial {\widetilde{L}}}{\partial t}=\nabla \cdot \left( d_{L}\left( x\right) \nabla {\widetilde{L}}\right) +\delta _{1}\left( x,t\right) {\widetilde{E}}+\delta _{2}\left( x,t\right) \widetilde{I_{1}} \\ \text { \ \ \ \ \ \ }-\left[ \alpha \left( x,t\right) +\theta \left( x,t\right) +\mu \left( x,t\right) \right] {\widetilde{L}}, \\ \frac{\partial I\widetilde{_{1}}}{\partial t}=\nabla \cdot \left( d_{I_{1}}\left( x\right) \nabla \widetilde{I_{1}}\right) +\gamma \left( x,t\right) {\widetilde{E}}+\theta \left( x,t\right) {\widetilde{L}}+\rho _{1}\left( x,t\right) {\widetilde{R}} \\ \text { \ \ \ \ \ \ }-\left[ \delta _{2}\left( x,t\right) +\omega \left( x,t\right) +\mu \left( x,t\right) +\eta _{1}\left( x,t\right) \right] \widetilde{I_{1}}, \\ \frac{\partial I_{2}}{\partial t}=\omega \left( x,t\right) \widetilde{I_{1}}- \left[ \phi \left( x,t\right) +\mu \left( x,t\right) +\eta _{2}\left( x,t\right) \right] \widetilde{I_{2}}, \\ \frac{\partial R}{\partial t}=\nabla \cdot \left( d_{R}\left( x\right) \nabla R\right) +\phi \left( x,t\right) \widetilde{I_{2}} \\ \text { \ \ \ \ \ \ }-\left[ \rho _{1}\left( x,t\right) +\rho _{2}\left( x,t\right) +\sigma \left( x,t\right) +\mu \left( x,t\right) +\eta _{3}\left( x,t\right) \right] {\widetilde{R}}, \\ \frac{\partial Q}{\partial t}\le \sigma \left( x,t\right) {\widetilde{R}}-\mu \left( x,t\right) {\widetilde{Q}}. \end{array}\right. } \end{aligned}$$
  By the comparison principle [18, Lemma 5.2.1], for every $$x\in \Omega$$ and $$t\ge 0$$,
  $$\begin{aligned} E\left( x,t\right)\le & {} {\widetilde{E}}\left( x,t\right) ,L\left( x,t\right) \le {\widetilde{L}}\left( x,t\right) ,I_{1}\left( x,t\right) \le \widetilde{ I_{1}}\left( x,t\right) ,I_{2}\left( x,t\right) \le \widetilde{I_{2}}\left( x,t\right) , \\ R\left( x,t\right)\le & {} {\widetilde{R}}\left( x,t\right) ,Q\left( x,t\right) \le {\widetilde{Q}}\left( x,t\right) . \end{aligned}$$
  Since $${\widetilde{E}}\left( x,t\right) \rightarrow 0,{\widetilde{L}}\left( x,t\right) \rightarrow 0,\widetilde{I_{1}}\left( x,t\right) \rightarrow 0, \widetilde{I_{2}}\left( x,t\right) \rightarrow 0,{\widetilde{R}}\left( x,t\right) \rightarrow 0,{\widetilde{Q}}\left( x,t\right) \rightarrow 0$$ as $$t\rightarrow \infty$$ for each $$x\in \Omega$$, we also have that
  $$\begin{aligned} I_{1}\left( x,t\right) \rightarrow 0,E\left( x,t\right) \rightarrow 0,L\left( x,t\right) \rightarrow 0,I_{2}\left( x,t\right) \rightarrow 0,R\left( x,t\right) \rightarrow 0,Q\left( x,t\right) \rightarrow 0 \end{aligned}$$
  as $$t\rightarrow \infty$$ for each $$x\in \Omega$$. Next we declare $$S\left( \cdot ,t\right) \rightarrow S^{0}\left( x\right)$$ uniformly on as $$t\rightarrow \infty$$. Given any small constant $$\varepsilon >0$$, there exists a large time $$T>0$$ such that $$0\le E\left( x,t\right) ,L\left( x,t\right) ,I_{1}(x,t)\le \varepsilon$$ for all $$x\in {\overline{\Omega }},t\ge T$$. From the first equation in system (1), it is noticed that *S* is a super-solution to
  5 $$\begin{aligned} \left\{ \begin{array}{l} \frac{\partial w}{\partial t}-\nabla \cdot \left( d_{S}\left( x\right) \nabla w\right) =\Lambda \left( x,t\right) -\left( \beta _{1}^{*}+\beta _{2}^{*}\right) w\varepsilon -\mu \left( x,t\right) w,\text { }x\in \Omega ,t\ge T, \\ \frac{\partial w}{\partial n}=0,\text { }x\in \partial \Omega , \\ w\left( x,T\right) =S\left( x,T\right) ,\text { }x\in \Omega \end{array} 
\right. \end{aligned}$$
  and a sub-solution to
  6 $$\begin{aligned} \left\{ \begin{array}{l} \frac{\partial v}{\partial t}-\nabla \cdot \left( d_{S}\left( x\right) \nabla v\right) =\Lambda \left( x,t\right) -\mu \left( x,t\right) v+\alpha \left( x,t\right) \varepsilon ,\text { }x\in \Omega ,t\ge T, \\ \frac{\partial v}{\partial n}=0,\text { }x\in \partial \Omega , \\ v\left( x,T\right) =S\left( x,T\right) ,\text { }x\in \Omega . \end{array} \right. \end{aligned}$$
  Denote by *w* and *v* the solution of system (5) and system (6), respectively. The parabolic comparison principle gives that
  $$\begin{aligned} w\left( x,t\right) \le S\left( x,t\right) \le v\left( x,t\right) \text { for all }x\in {\overline{\Omega }},t\ge T\text {.} \end{aligned}$$
  For system (5), we can verify that
  $$\begin{aligned}&\left\langle \nabla \cdot \left( d_{S}\left( x\right) \nabla w\right) +\Lambda \left( x,t\right) -\left( \beta _{1}^{*}+\beta _{2}^{*}\right) w\varepsilon -\mu \left( x,t\right) w,w\right\rangle _{{\mathbf {H}}} \\ =&\int _{\Omega }\nabla \cdot \left( d_{S}\left( x\right) \nabla w\right) \cdot wdx+\int _{\Omega }\Lambda \left( x,t\right) wdx \\&-\int _{\Omega }\left( \beta _{1}^{*}+\beta _{2}^{*}\right) \varepsilon w^{2}dx-\int _{\Omega }\mu \left( x,t\right) w^{2}dx \\ \le&-\left( d_{S}\right) _{*}\left\| w\right\| _{{\mathbf {H}}_{ \frac{1}{2}}}^{2}+\Lambda ^{*}\int _{\Omega }wdx, \end{aligned}$$
  this means that system (5) satisfies [22, condition (2.3)] for $$\mathcal {L}w+G\left( w\right) =\nabla \cdot \left( d_{S}\left( x\right) \nabla w\right) +\Lambda \left( x,t\right) -\left( \beta _{1}^{*}+\beta _{2}^{*}\right) w\varepsilon -\mu \left( x,t\right) w$$. Same as the proof of Theorem 3 in the previous article, system (5) also exists a global exponential attractor $${\mathcal {A}}_{w}$$. In addition, system (5) has a variational structure, the corresponding functional of the variational structure is
  $$\begin{aligned} F\left( w\right) =\int _{\Omega }\left[ \frac{d_{S}\left( x\right) }{2} \left| \nabla w\right| ^{2}-g\left( x,w\right) \right] dx, \end{aligned}$$
  where
  $$\begin{aligned} g\left( x,w\right) =\int _{0}^{w}\left[ \Lambda \left( x,t\right) -\left( \beta _{1}^{*}+\beta _{2}^{*}\right) w\varepsilon -\mu \left( x,t\right) w\right] du. \end{aligned}$$
  Then
  $$\begin{aligned}&\left\langle DF\left( w\right) ,{\mathcal {L}}w+G\left( w\right) \right\rangle _{ {\mathbf {H}}} \\ =&\left\langle DF\left( w\right) ,\nabla \cdot \left( d_{S}\left( x\right) \nabla w\right) +\Lambda \left( x,t\right) -\left( \beta _{1}^{*}+\beta _{2}^{*}\right) w\varepsilon -\mu \left( x,t\right) w\right\rangle _{ {\mathbf {H}}} \\ =&-\left\| DF\left( w\right) \right\| _{{\mathbf {H}}}^{2}, \end{aligned}$$
  so $${\mathcal {L}}+G$$ is a gradient type operator. From [12, Theorem A.2.2], we can prove that
  $$\begin{aligned} \underset{t\rightarrow \infty }{\lim }w\left( x,t\right) =S_{-}^{0}\left( \varepsilon ,x\right) \text { in }{\mathbf {H}}\text {,} \end{aligned}$$
  where $$S_{-}^{0}\left( \varepsilon ,x\right)$$ is the unique positive steady state of problems (5). Similarly, for system (6), we can obtain
  $$\begin{aligned} \underset{t\rightarrow \infty }{\lim }v\left( x,t\right) =S_{+}^{0}\left( \varepsilon ,x\right) \text { in }{\mathbf {H}}\text {,} \end{aligned}$$
  where $$S_{+}^{0}\left( \varepsilon ,x\right)$$ is the unique positive steady state of problems (6). Furthermore, because of the arbitrariness of $$\varepsilon$$, it is easily checked that
  $$\begin{aligned} S_{-}^{0}\left( \varepsilon ,x\right) ,S_{+}^{0}\left( \varepsilon ,x\right) \rightarrow S^{0}\left( x\right) \text { in }{\mathbf {H}}\text {, as } \varepsilon \rightarrow 0\text {.} \end{aligned}$$
  Thus, our analysis implies that the $$S\left( \cdot ,t\right) \rightarrow S^{0}\left( x\right)$$ uniformly as $$t\rightarrow \infty$$. In this way, we have proved that when $$t\rightarrow \infty$$, without any form of infection, the COVID-19 epidemic has completely disappeared.
2. Since $$\uplambda ^{*}>0$$, it is noticed that the solution of
  7 $$\begin{aligned} \left\{ \begin{array}{l} \frac{\partial S_{-}}{\partial t}-\nabla \cdot \left( d_{S}\left( x\right) \nabla S_{-}\right) =\Lambda \left( x,t\right)-\left[ \beta _{1}\left( x,t\right) K^{*}+\beta _{1}\left( x,t\right) K^{*}+\mu \left( x,t\right) \right] S_{-}, \\ \frac{\partial S_{-}}{\partial n}=0,\text { }x\in \partial \Omega , \\ S_{-}\left( x,T\right) =S\left( x,T\right) ,\text { }x\in \Omega \end{array} \right. \end{aligned}$$
  is a sub-solution of the first equation in novel coronavirus pneumonia transmission model (1). Similar to the proof of conclusion (1), system (7) is also a gradient type equation. From [12, Theorem A.2.2], we can prove that
  $$\begin{aligned} \underset{t\rightarrow \infty }{\lim }S_{-}\left( x,t\right) =S_{-}^{*}\left( x\right) \text { in }{\mathbf {H}}\text {.} \end{aligned}$$
  By weak maximum principle, we know that $$S_{-}^{*}\left( x\right) >0$$ for all $$x\in \Omega$$. Define that
  $$\begin{aligned}&\left( E_{-}\left( x,t\right) ,L_{-}\left( x,t\right) ,I_{1-}\left( x,t\right) ,I_{2-}\left( x,t\right) ,R_{-}\left( x,t\right) ,Q_{-}\left( x,t\right) \right) \\= & {} \left( \varepsilon \chi ^{*}\left( x\right) ,\varepsilon \kappa ^{*}\left( x\right) ,\varepsilon \varphi ^{*}\left( x\right) ,\varepsilon \psi ^{*}\left( x\right) ,\varepsilon \xi ^{*}\left( x\right) ,\varepsilon \zeta ^{*}\left( x\right) \right) \end{aligned}$$
  and
  $$\begin{aligned} S^{0}=S\pm \varepsilon \vartheta ^{*}\left( x\right) \ge S_{-}^{*}\left( x\right) >0 \end{aligned}$$
  where
  $$\begin{aligned} \begin{array}{l} \vartheta ^{*}\left( x\right)>>0,\chi ^{*}\left( x\right)>>0,\kappa ^{*}\left( x\right)>>0,\varphi ^{*}\left( x\right)>>0, \\ \psi ^{*}\left( x\right)>>0,\xi ^{*}\left( x\right)>>0,\zeta ^{*}\left( x\right)>>0 \end{array} \end{aligned}$$
  and $$\varepsilon >0$$ is a sufficiently small constant. Substituting $$\varepsilon \chi ^{*}\left( x\right) ,\varepsilon \kappa ^{*}\left( x\right) ,\varepsilon \varphi ^{*}\left( x\right) ,\varepsilon \psi ^{*}\left( x\right) ,\varepsilon \xi ^{*}\left( x\right) ,\varepsilon \zeta ^{*}\left( x\right)$$ into the second, the third, the forth, the fifth and the sixth equations of system (1), we know
  $$\begin{aligned}&\frac{1}{S^{0}}\left\{ \varepsilon \nabla \cdot \left( d_{E}\left( x\right) \nabla \chi ^{*}\right) +\beta _{1}\left( x,t\right) S\varepsilon \chi ^{*}+\beta _{2}\left( x,t\right) S\varphi ^{*}+\rho _{2}\left( x,t\right) \varepsilon \xi ^{*}\right. \\&\left. -\left[ \delta _{1}\left( x,t\right) +\gamma \left( x,t\right) +\mu \left( x,t\right) \right] \varepsilon \chi ^{*}-\frac{\partial \left( \varepsilon \chi ^{*}\right) }{\partial t}\right\} \\ =&\frac{1}{S^{0}}\left\{ \varepsilon \nabla \cdot \left( d_{E}\left( x\right) \nabla \chi ^{*}\right) +\beta _{1}\left( x,t\right) S^{0}\varepsilon \chi ^{*}+\beta _{2}\left( x,t\right) S^{0}\varepsilon \varphi ^{*}+\rho _{2}\left( x,t\right) \varepsilon \xi ^{*}\right. \\&-\left[ \delta _{1}\left( x,t\right) +\gamma \left( x,t\right) +\mu \left( x,t\right) \right] \varepsilon \chi ^{*}+\beta _{1}\left( x,t\right) S\varepsilon \chi ^{*}+\beta _{2}\left( x,t\right) S\varepsilon \varphi ^{*} \\&\left. -\beta _{1}\left( x,t\right) S^{0}\varepsilon \chi ^{*}-\beta _{2}\left( x,t\right) S^{0}\varepsilon \varphi ^{*}\right\} \\ =&\frac{1}{S^{0}}\varepsilon \uplambda ^{*}\varphi ^{*}+\beta _{1}\left( x,t\right) \varepsilon \chi ^{*}\left[ \frac{S}{S^{0}}-1 \right] +\beta _{2}\left( x,t\right) \varepsilon \varphi ^{*}\left[ \frac{S}{S^{0}}-1\right] \\ =&\frac{1}{S^{0}}\varepsilon \uplambda ^{*}\varphi ^{*}+\varepsilon \left( \beta _{1}\left( x,t\right) \chi ^{*}+\beta _{2}\left( x,t\right) \varphi ^{*}\right) \left[ \frac{S}{S\pm \varepsilon \vartheta ^{*}\left( x\right) }-1\right]>0\text { } \\ \text {(}\varepsilon&>0\text { is a sufficiently small constant),} \end{aligned}$$
  $$\begin{aligned}&\varepsilon \nabla \cdot \left( d_{L}\left( x\right) \nabla \kappa ^{*}\right) +\delta _{1}\left( x,t\right) \varepsilon \chi ^{*}+\delta _{2}\left( x,t\right) \varepsilon \varphi ^{*} \\&-\left[ \alpha \left( x,t\right) +\theta \left( x,t\right) +\mu \left( x,t\right) \right] \varepsilon \kappa ^{*}-\frac{\partial \left( \varepsilon \kappa ^{*}\right) }{\partial t} \\ =&\varepsilon \uplambda ^{*}\kappa ^{*}>0\text { (}\varepsilon >0\text { is a sufficiently small constant),} \end{aligned}$$
  $$\begin{aligned}&\varepsilon \nabla \cdot \left( d_{I_{1}}\left( x\right) \nabla \varphi ^{*}\right) +\gamma \left( x,t\right) \varepsilon \chi ^{*}+\theta \left( x,t\right) \varepsilon \kappa ^{*}+\rho _{1}\left( x,t\right) \varepsilon \xi ^{*} \\&-\left[ \delta _{2}\left( x,t\right) +\omega \left( x,t\right) +\mu \left( x,t\right) +\eta _{1}\left( x,t\right) \right] \varepsilon \varphi ^{*}- \frac{\partial \left( \varepsilon \varphi ^{*}\right) }{\partial t} \\ =&\varepsilon \uplambda ^{*}\psi ^{*}>0\text { (}\varepsilon >0\text { is a sufficiently small constant),} \end{aligned}$$
  $$\begin{aligned}&\omega \left( x,t\right) \varepsilon \varphi ^{*}-\left[ \phi \left( x,t\right) +\mu \left( x,t\right) +\eta _{2}\left( x,t\right) \right] \varepsilon \psi ^{*}-\frac{\partial \left( \varepsilon \psi ^{*}\right) }{\partial t} \\ =&\varepsilon \uplambda ^{*}\psi ^{*}>0\text { (}\varepsilon >0\text { is a sufficiently small constant),} \end{aligned}$$
  $$\begin{aligned}&\varepsilon \nabla \cdot \left( d_{R}\left( x\right) \nabla \xi ^{*}\right) +\phi \left( x,t\right) \psi ^{*}-\frac{\partial \left( \varepsilon \xi ^{*}\right) }{\partial t} \\&-\left[ \rho _{1}\left( x,t\right) +\rho _{2}\left( x,t\right) +\sigma \left( x,t\right) +\mu \left( x,t\right) +\eta _{3}\left( x,t\right) \right] \varepsilon \xi ^{*} \\ =&\varepsilon \uplambda ^{*}\xi ^{*}>0\text { (}\varepsilon >0\text { is a sufficiently small constant)} \end{aligned}$$
  and
  $$\begin{aligned}&\sigma \left( x,t\right) \varepsilon \xi ^{*}-\mu \left( x,t\right) \varepsilon \zeta ^{*}-\frac{\partial \left( \varepsilon \zeta ^{*}\right) }{\partial t} \\ =&\varepsilon \uplambda ^{*}\zeta ^{*}>0\text { (}\varepsilon >0\text { is a sufficiently small constant)} \end{aligned}$$
  Therefore, $$\left( \varepsilon \chi ^{*},\varepsilon \kappa ^{*},\varepsilon \varphi ^{*},\varepsilon \psi ^{*},\varepsilon \xi ^{*},\varepsilon \zeta ^{*}\right)$$ is the sub-solution of the second, the third, the forth and the fifth equations of system (1). We choose $$0<\varrho \left( x\right) <\min \left\{ S_{-}^{*}\left( x\right) ,\varepsilon \chi ^{*}\left( x\right) ,\varepsilon \kappa ^{*}\left( x\right) ,\varepsilon \varphi ^{*}\left( x\right) ,\varepsilon \psi ^{*}\left( x\right) ,\varepsilon \xi ^{*}\left( x\right) ,\varepsilon \zeta ^{*}\left( x\right) \right\}$$, we can obtain that
  $$\begin{aligned} \underset{t\rightarrow \infty }{\lim \inf }S\left( x,t\right)\ge & {} \varrho \left( x\right) ,\underset{t\rightarrow \infty }{\lim \inf }I_{1}\left( x,t\right) \ge \varrho \left( x\right) ,\underset{t\rightarrow \infty }{ \lim \inf }I_{2}\left( x,t\right) \ge \varrho \left( x\right) , \\ \underset{t\rightarrow \infty }{\lim \inf }R\left( x,t\right)\ge & {} \varrho \left( x\right) ,\underset{t\rightarrow \infty }{\lim \inf }Q\left( x,t\right) \ge \varrho \left( x\right) \end{aligned}$$
  for $$x\in {\overline{\Omega }}$$, then it shows that the novel coronavirus pneumonia persists.

$$\square$$

The results of Theorem 2.4 show that $\uplambda ^{*}$ is a threshold for describing the infectious ability of novel coronavirus pneumonia. If $\uplambda ^{*}>0$ and $t\rightarrow \infty$, then the positive solution of novel coronavirus pneumonia transmission model (1) is globally exponential attractive and the attraction domain is ${\mathcal {A}}^{*}$. To explain this phenomenon from a medical point of view is that the novel coronavirus pneumonia epidemic will continue to survive and cannot be cured, but the spread of the epidemic will eventually be effectively controlled within a small area.

### Data collection and analysis

The number of confirmed COVID-19 cases worldwide has exceeded 100 million, and the prevention and control of the epidemic is still very arduous. As the northern hemisphere enters winter, epidemics in many countries in Europe and the United States have broken out again, and confirmed cases have increased day by day.

From January 24, 2020, the official website of the National Health Committee of the People’s Republic of China has updated the relevant data of the COVID-19 epidemic [13]. The official website of the WHO can check the relevant data from January 21 to the present [8]. Our data comes from these official websites, and the data on the website will be updated in a timely manner every day. Based on these data, we can get some important parameters in Table 2 through simple data analysis and calculations.

**Table 2:** The parameters description of the COVID-19 epidemic in China

Parameter	Data estimated	Data sources
$\Lambda$	5	Estimate
$\beta _1$	0.6	References [23]
$\beta _2$	0.3	References [23]
$\alpha$	0.3	Estimate
$\gamma +\delta _2$	0.423	References [13]
$\delta _1$	0.5	Estimate
$\omega$	0.35	References [13]
$\rho _1$	0.001	References [23]
$\rho _2$	0.002	References [23]
$\theta$	0.7	Estimate
$\phi$	0.8	References [13]
$\sigma$	0.7	Estimate
$\mu$	0.1595	References [19]
$\eta _{1}$	0.021	References [13]
$\eta _{2}$	0.157	References [13]
$\eta _{3}$	0.021	References [13]
$\tau$	11	References [23]

### Numerical simulation of novel coronavirus pneumonia epidemic trend in China

Since the outbreak of COVID-19 in 2020, the global spread of the epidemic has shown a certain periodicity, and this periodic phenomenon is the result of a combination of time and space factors. First of all, the diffusion of the epidemic is highly dependent on climate and temperature, and the root cause of temperature differences between regions is the difference in latitude and location, and it is finally manifested through the time phenomenon of seasonal alternation. In daily life, people’s activity trajectories are regularly fixed between several specific locations, such as homes, work units, schools, subways, supermarkets, and so on. The appearance of these specific locations in daily life is also periodic and the diffusion rate of each location is relatively fixed. Therefore, the position in the trajectory of people’s action will appear periodically, and the diffusion rate will also appear periodically with this trajectory of action. In order to investigate the impact of the spatial periodic diffusion rate on the spread of the epidemic, we select a positive periodic function according to the range of activities of different groups of people (the susceptible people have a large range of activities, and the infected people have a small range of activities). Through a number of numerical simulation experiments, we found that the simulation effect of the set of parameters $d_{S}\left( x\right) =e^{15\sin x}, d_{E}\left( x\right) =\left| \sin x\right| , d_{L}\left( x\right) =\left| \sin x\right| , d_{I_{1}}\left( x\right) =0.3\left| \sin x\right| , d_{R}\left( x\right) =2$ is more consistent with the actual spread of the epidemic.

**Fig. 2:**
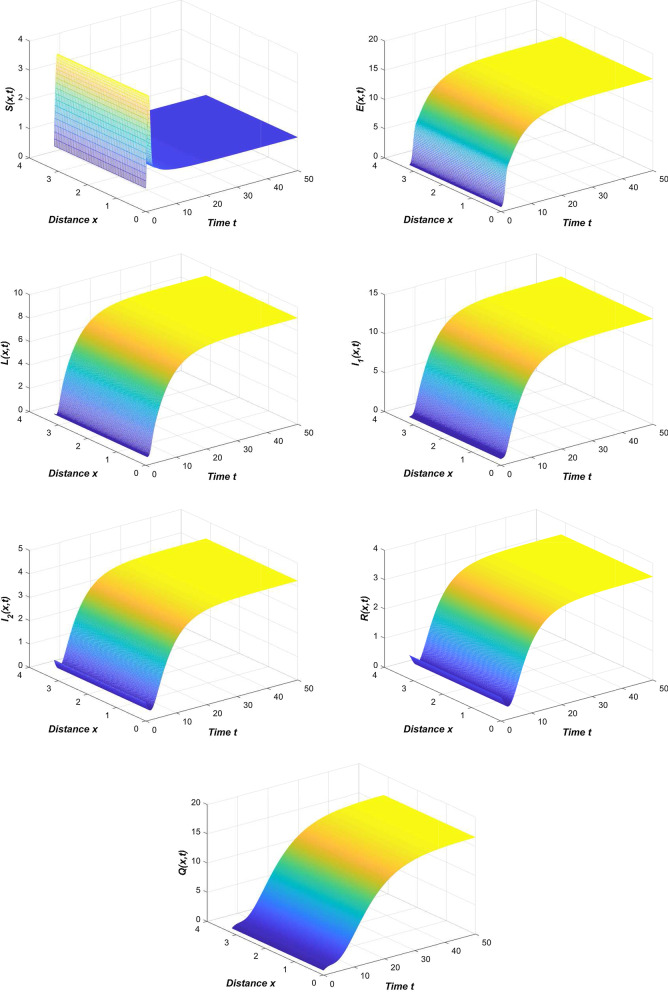
The spread of the COVID-19 epidemic in China

Refer to the data in Table 2 and our system (1), we first simulate the spread trend of the novel coronavirus pneumonia epidemic in China (Fig. 2).

From Fig. 2, we can see that the results of the numerical simulation are basically consistent with the official data. At this time, the novel coronavirus pneumonia epidemic is globally asymptotically stable or persists uniformly.

**Fig. 3:**
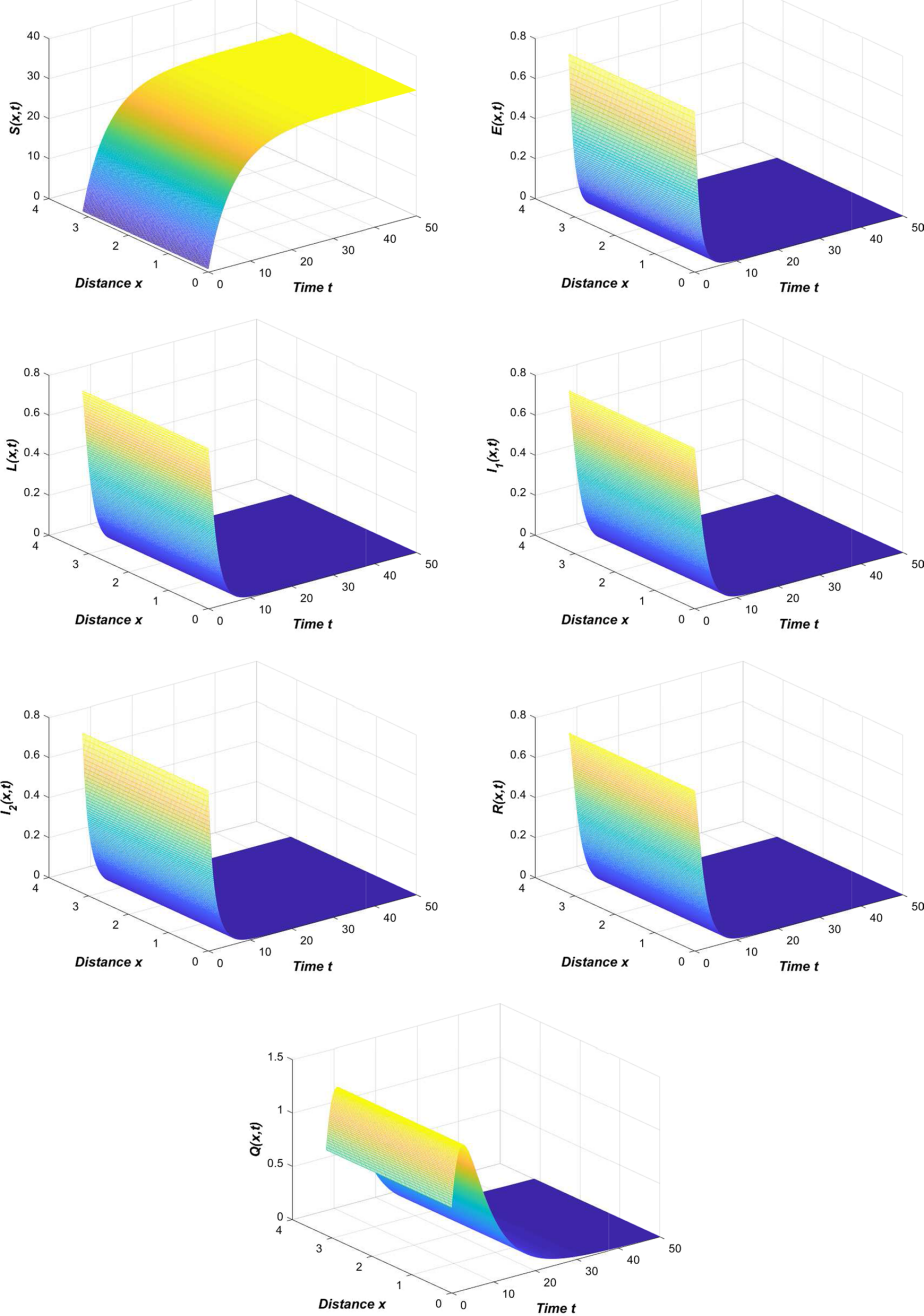
The spread of the COVID-19 epidemic when $\beta _1=0.006, \beta _2=0.003$

If we choose $\beta _1=0.006,\beta _2=0.003$ in Table 2, then we can obtain the image in Fig. 3.

From Fig. 3, we find that when the contact rate is reduced to a small enough level, the novel coronavirus pneumonia epidemic will die out. At this time, the disease-free equilibrium is globally asymptotically stable.

**Fig. 4:**
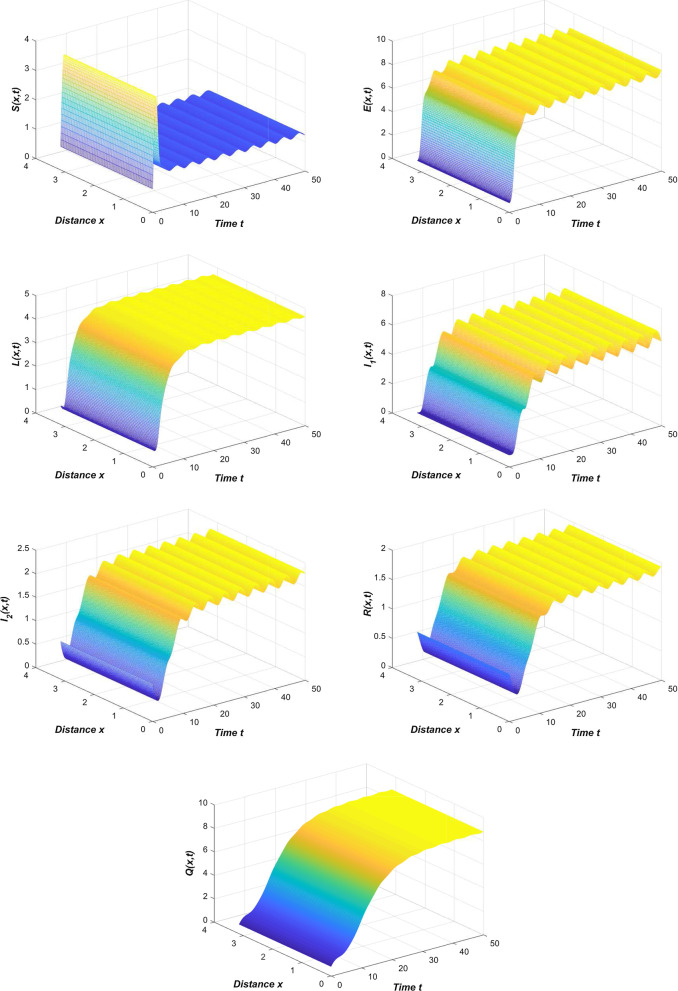
The temporal-spatial heterogeneity COVID-19 epidemic is persists uniformly when $\beta _{1}\left( x,t\right) =0.3\left| 0.2\sin x\right| ,\gamma \left( x,t\right) =0.223\left| \cos x\right| ,\rho _{2}\left( x,t\right) =0.02\left| \sin x\right| ,\eta _{2}\left( x,t\right) =0.157e^{-2xt}$

From the novel coronavirus pneumonia transmission model (1) we can see that all the parameters are temporal-spatial related functions, so we choose different functions will directly lead to different stability results. If we select ${\beta _{1}}\left( x,t\right) =0.3\left| 0.2\sin x\right| ,{\gamma } \left( x,t\right) =0.223\left| \cos x\right| ,{\rho _{2}}\left( x,t\right) =0.02\left| \sin x\right| ,{\eta _{2}}\left( x,t\right) =0.157e^{-2xt}$ and choose other parameters from Table 2, then we can clearly see that the novel coronavirus pneumonia epidemic is persists uniformly (Fig. 4). This reflects that the new coronavirus epidemic will fluctuate within a controllable range, but the epidemic will not dissipate. This is the normalization stage of the spread of COVID-19.

**Fig. 5:**
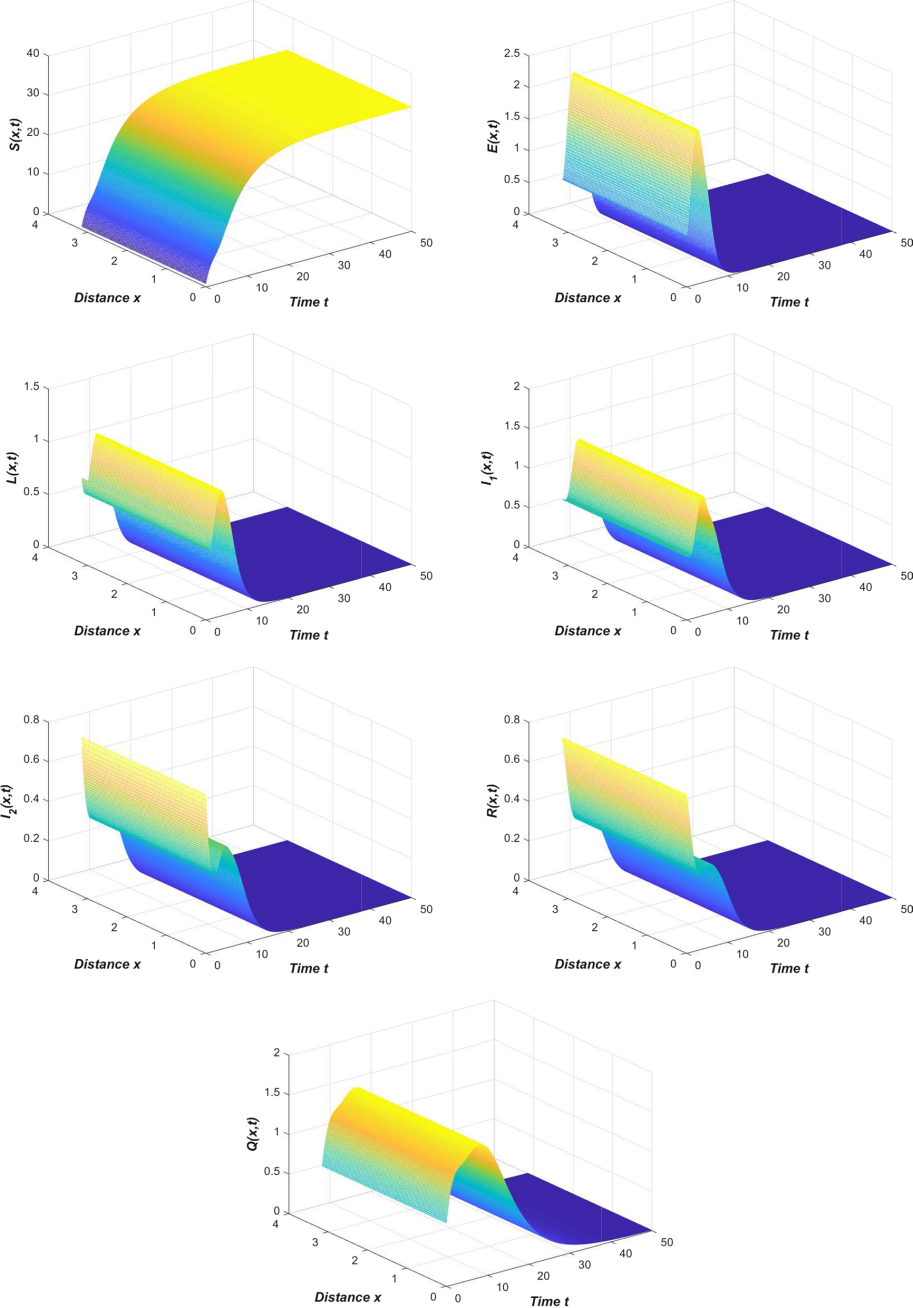
The global stability of disease-free equilibrium of temporal-spatial heterogeneity system (1) when $\beta _{1} \left( x,t\right) =0.6e^{-x},\beta _{2}\left( x,t\right) =0.3e^{-2x}\left| \sin xt\right| ,\gamma \left( x,t\right) =0.223\left| \cos x\right| ,\rho _{2}\left( x,t\right) =0.02\left| \sin x\right| ,\eta _{2}\left( x,t\right) =0.157e^{-2xt}$

If we select ${\beta _{1}}\left( x,t\right) =0.6e^{-x},{\beta _{2}}\left( x,t\right) =0.3e^{-2x}\left| \sin xt\right| ,\gamma \left( x,t\right) =0.223\left| \cos x\right| , {\rho _{2}}\left( x,t\right) =0.02\left| \sin x\right| ,{\eta _{2}}\left( x,t\right) =0.157e^{-2xt}$ and choose other parameters from Table 2, then we can clearly see that the disease-free equilibrium of the temporal-spatial heterogeneity novel coronavirus pneumonia epidemic is globally asymptotically stable (Fig. 5).

## Discussion

The novel coronavirus pneumonia epidemic is still raging around the world. As of February 3, 2020, the five most severely affected countries in the world are the United States, India, Brazil, the United Kingdom and Russia. As a populous country, China has done a very good job in the prevention and control of the novel coronavirus pneumonia epidemic, with only sporadic cases of asymptomatic infections and imported cases from abroad. We make a list of the real-time data of the above several countries for comparison in Table 3.

**Table 3:** The number of confirmed cases and deaths of COVID-19 in China and the five most severe countries

Country	Total confirmed cases	Newly confirmed cases	Total deaths
USA	27027347	114703	457856
India	10766245	8635	154486
Brazil	9283418	54096	226309
UK	3863757	16906	108225
Russia	3842145	16406	75383
China	101092	12	4828

Why is the prevention and control of the novel coronavirus pneumonia epidemic in China so effective? A very important point is that the Chinese government encourages people to take the initiative to stay at home and reduce gathering activities. The Chinese people also consciously wear masks when they go out and keep a safe distance from each other. The primary purpose of home quarantine measure is to control the effective contact rate, and it can also reduce the chance of relapse in patients after cures.

**Fig. 6:**
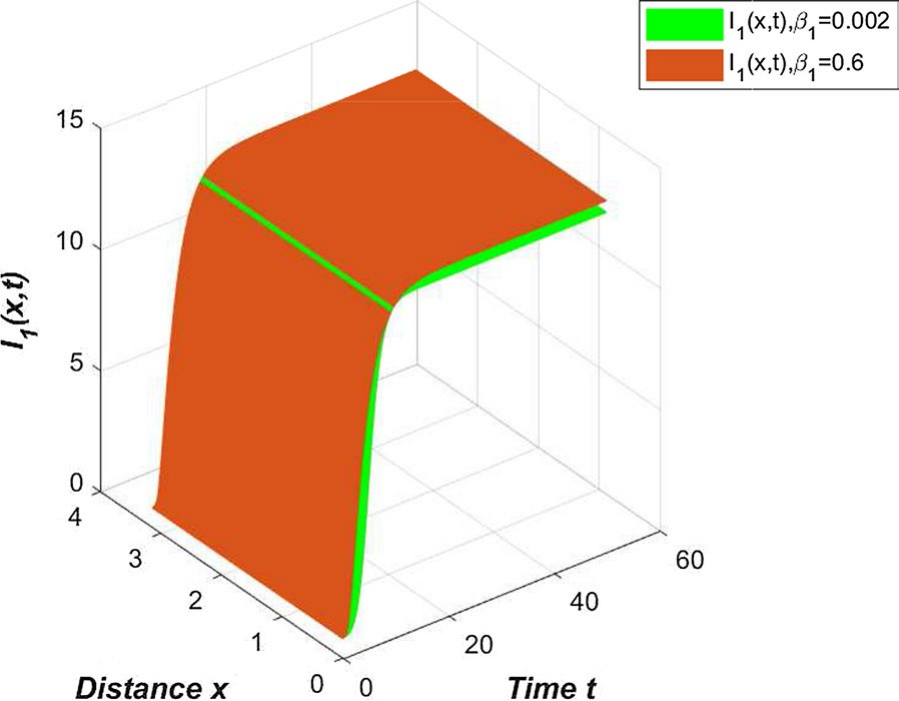
Comparison of compartment $I_{1}$ under different contact rates

In our model (1), compartment *E* contains asymptomatic infections and patients in the incubation period. These two groups of people cannot know that they are carrying the virus without medical treatment. They live a normal life like everyone else and can move around without restriction. Their dedication to spreading the new coronavirus is higher than the confirmed cases. We only adjust the contact rate $\beta _1=0.002$ in Table 2 to draw a comparison chart of compartment $I_{1}$ (Fig. 6).

From Fig. 6, we can clearly see that if there are ways to quickly identify asymptomatic infections and patients in the incubation period, and reduce contact with these people, China’s epidemic prevention and control can still do better.

**Fig. 7:**
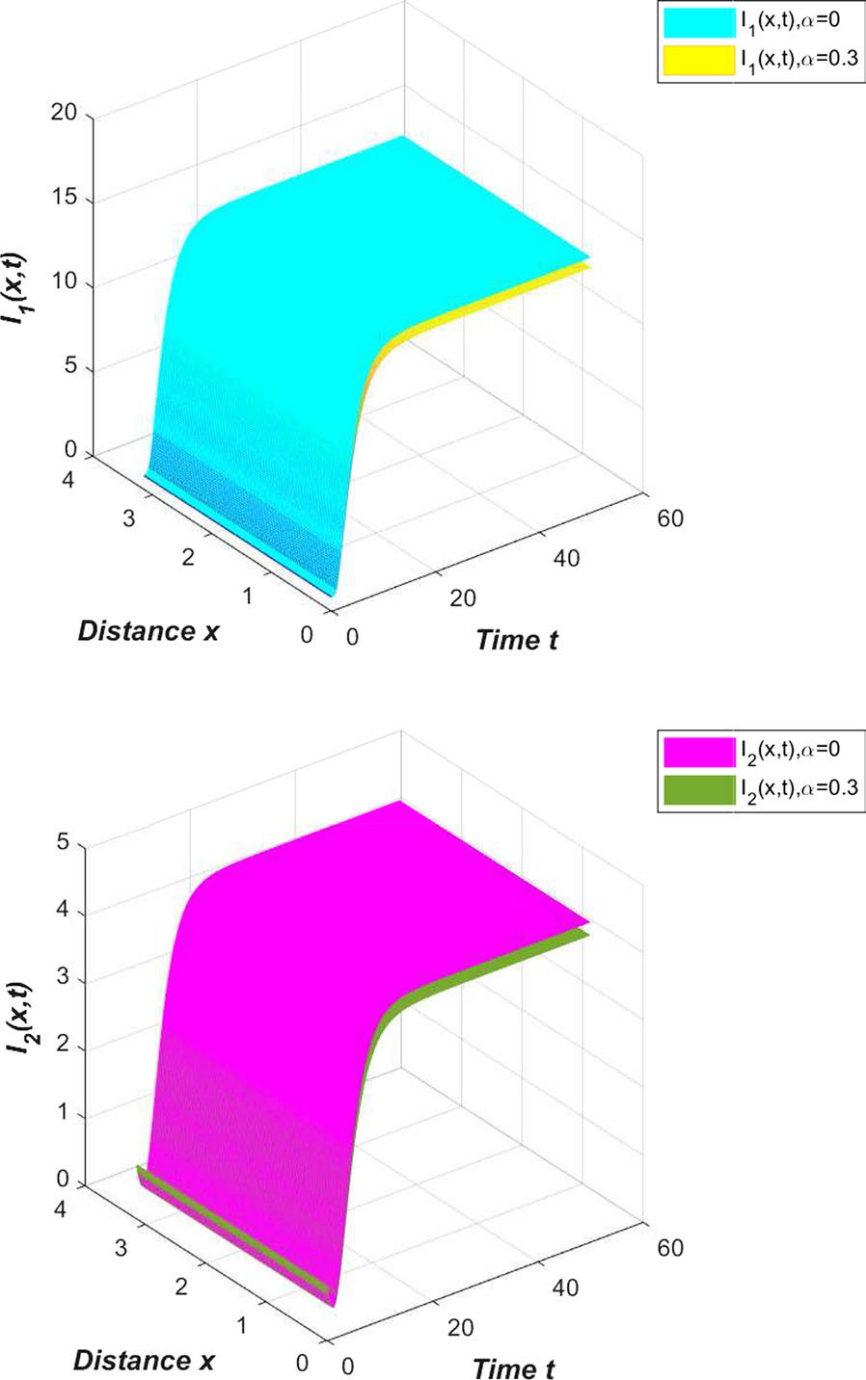
Effect of self-limiting treatment

Another highlight of the novel coronavirus pneumonia model constructed in this article is to examine the impact of self-limiting treatment on epidemic prevention and control. As mentioned earlier, self-limiting treatment includes symptomatic treatment, immunotherapy and other methods. Reasonable diet and strengthening exercise are all ways to enhance physical fitness and immunity. The successful development of the new crown vaccine has also greatly improved the immunity of the vaccinated population, enhanced the resistance of the vaccinated population and the self-healing ability after infection. Regarding the effect of self-limiting treatment, we conducted the following simulation. First, we simulate the number of infected people in China without any self-limiting treatment (Fig. 7).

**Fig. 8:**
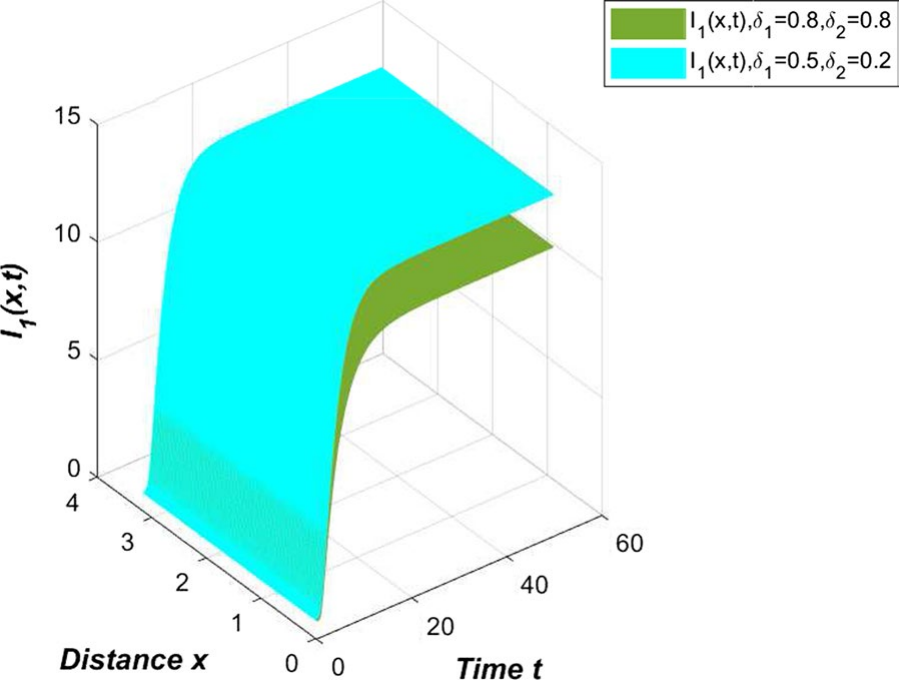
Comparison of compartment $I_{1}$ under different self-limiting treatment rates

From Fig. 7 we can clearly see that without vaccines, home isolation and other self-limiting treatment measures, the number of confirmed cases in China will be greater than the current number. The COVID-19 vaccine has been successfully developed in China, and the popularization of the vaccine has also begun. In the future, more and more people will participate in the immunotherapy of vaccination. If we adjust the self-limiting treatment rate such that $\delta _1=0.8,\delta _2=0.8$ in Table 2, the number of people participating in self-limiting treatment has increased significantly at this time, and we can see from Fig. 8 that the decline in infected people is even more obvious. Compared with $\delta _1=0.5,\delta _2=0.2$, the number of infections dropped by about $\frac{1}{3}$.

**Fig. 9:**
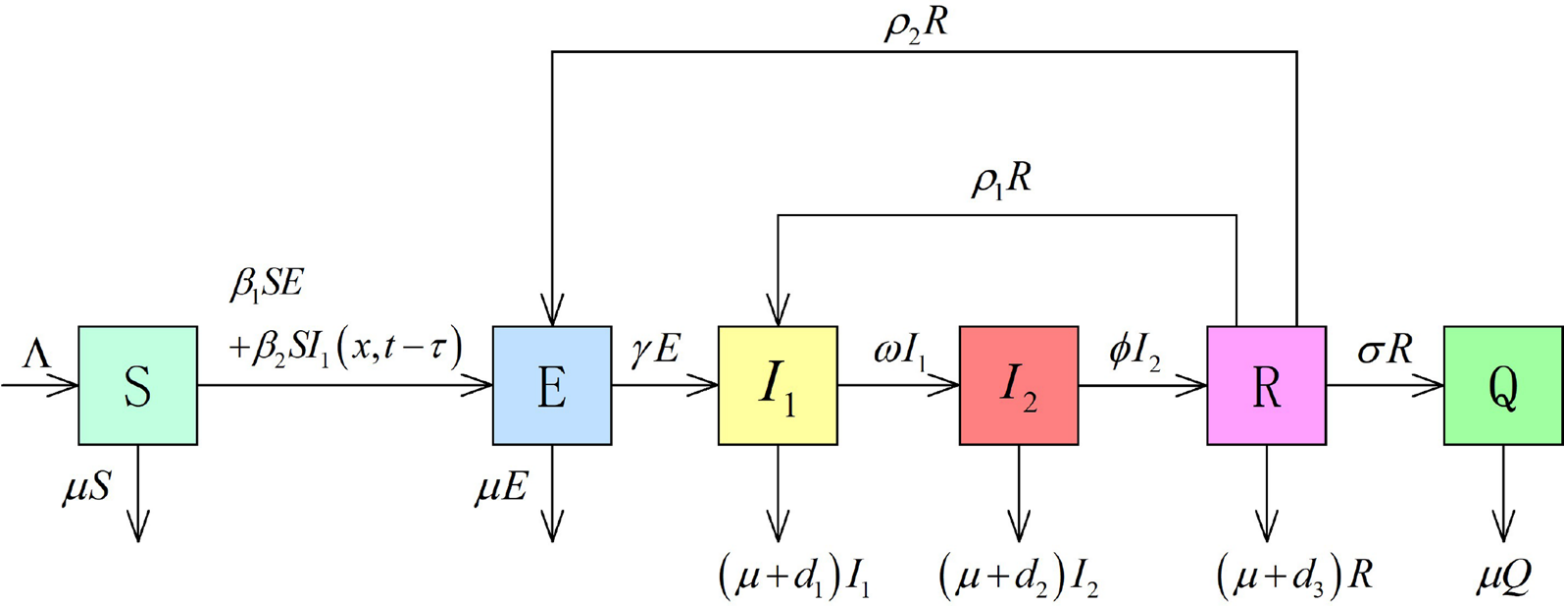
The COVID-19 model (1) missing the self-limiting treatment compartment

**Fig. 10:**
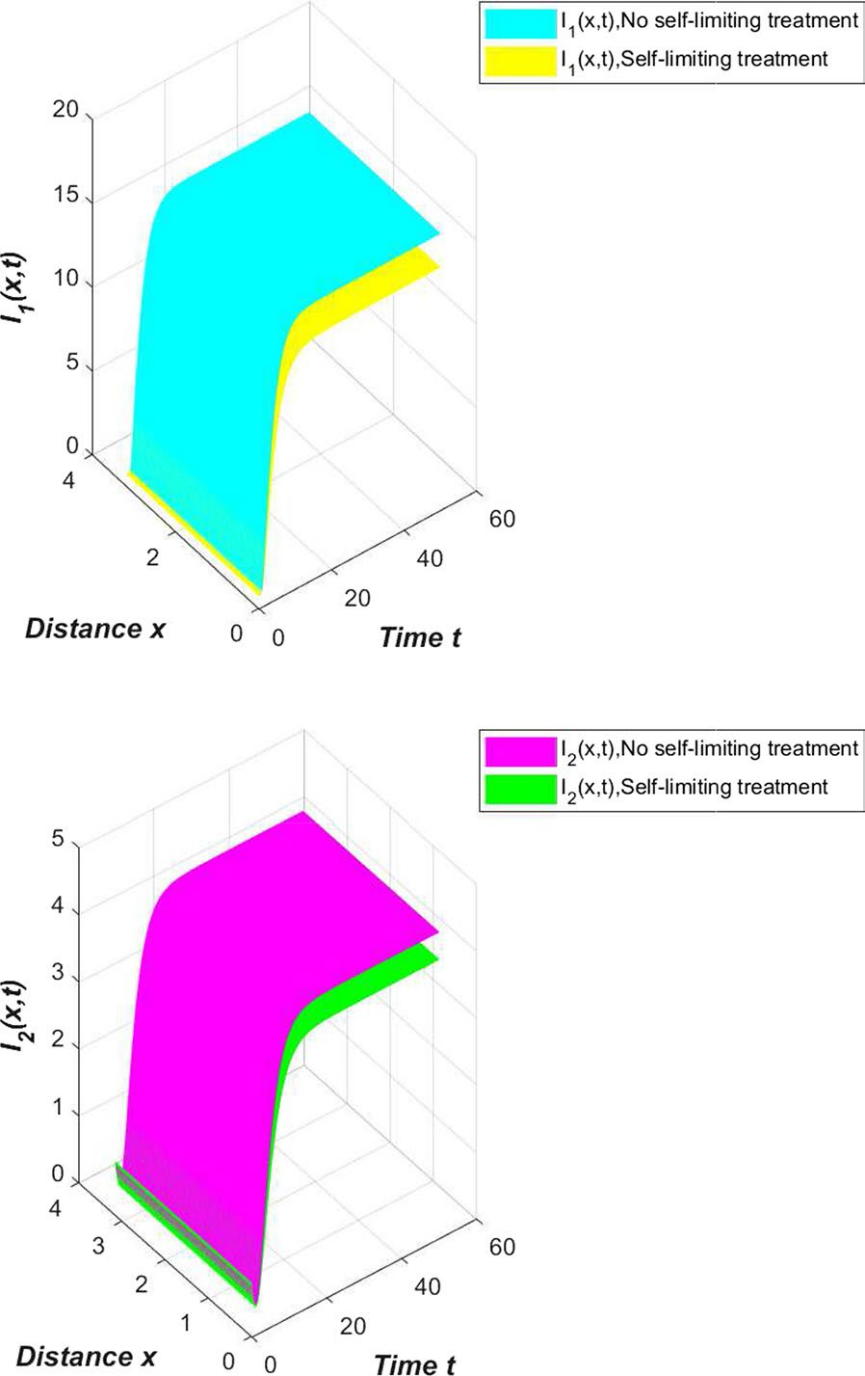
Comparison of the impact of self-limiting treatment on infected patients

If we choose $\alpha =\theta =\delta _{1}=\delta _{2}=0$ in Table 2, the self-limiting treatment compartment (*L*) in model (1) will be gone, and replaced by the following new model:

We still use the data in Table 2 to simulate the new model in Fig. 9, and compare the number of infected persons in the new model and model 0.1 to get the following comparison chart (Fig. 10).

Figure 10 clearly reflects the role of self-limiting treatment in epidemic prevention and control. If there is no self-limiting treatment compartment in the model, the number of infected persons is significantly higher than when there has self-limiting treatment. Compared with official data, the model without a self-limiting treatment compartment has larger errors in the simulation results. Therefore, the model (1) constructed in this article is more suitable for the spread of the epidemic in China. Prevention and control recommendations based on this model will also be more helpful to public health departments.

**Fig. 11:**
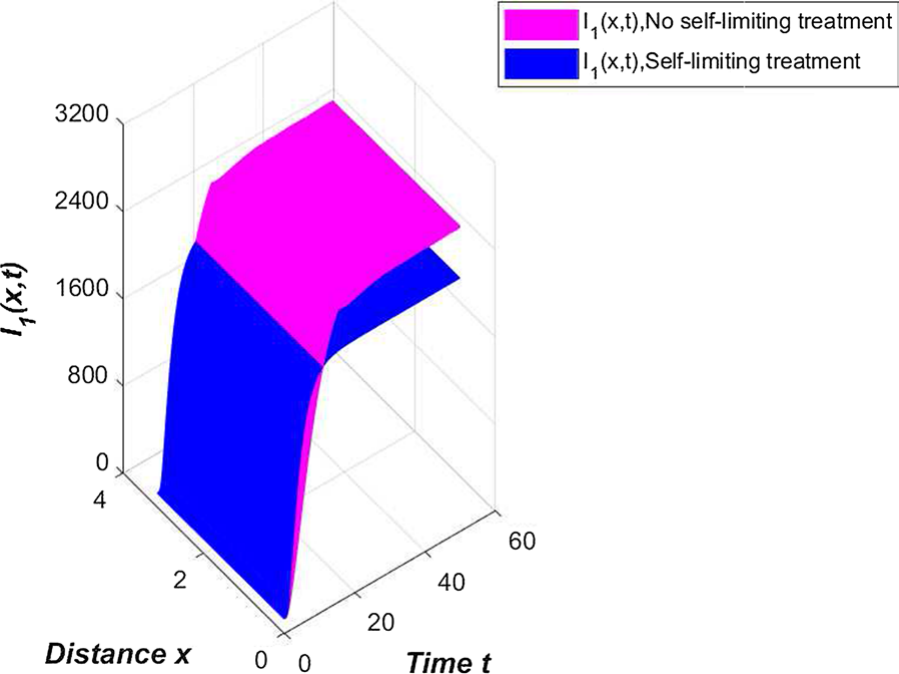
Comparison of US confirmed cases with or without self-limiting treatment

From Table 3, we can see that the cumulative number of confirmed cases in the United States has exceeded 27 million, which is a very alarming number. The Centers for Disease Control and Prevention publishes weekly summary of the novel coronavirus pneumonia epidemic in the USA [9]. The weekly summary shows that the incidence of the USA epidemic has dropped to 8.8%, the mortality rate has dropped to 5.5%, and the rate of isolation treatment in hospitals is 107.2/100000. Then we get the following data in Table 4. Since the outbreak of the epidemic, the daily life of the American people has not been subject to any restrictions. They work normally, gather together and lack the necessary protective measures. If the U.S. government encourages people to reduce going out, wear masks to travel, and take measures to isolate and self-limit the treatment of mild patients, the number of confirmed cases in the United States will be greatly reduced. Combined with the data in Table 4, we make a simulation comparison of confirmed cases in the United States with or without self-limiting treatment (Fig. 11). Obviously, self-limiting treatment can help the United States better prevent and control the novel coronavirus pneumonia epidemic.

**Table 4:** The parameters description of the COVID-19 epidemic in the USA

Parameter	Data estimated	Data sources
$\Lambda$	18000000	References [23]
$\beta _1$	0.75	References [23]
$\beta _2$	0.6	References [23]
$\gamma$	0.088	References [9]
$\omega$	0.001072	References [9]
$\rho _1$	0.1	References [23]
$\rho _2$	0.2	References [23]
$\phi$	0.35	References [23]
$\sigma$	0.55	Estimate
$\mu$	0.1595	References [19]
$\eta _{1}$	0.055	References [9]
$\eta _{2}$	0.055	References [9]
$\eta _{3}$	0.049	References [8]
$d_{S}$	2	Estimate
$d_{E}$	1	Estimate
$d_{I}$	0.3	Estimate
$d_{R}$	2	Estimate

Through the numerical simulations in this section, we find that restricting the free movement of asymptomatic infected persons can reduce the risk of infection for susceptible persons. Increasing the proportion of self-limiting treatment for asymptomatic infections and patients in the incubation period has a significant effect on the prevention and control of the novel coronavirus pneumonia epidemic.

**Fig. 12:**
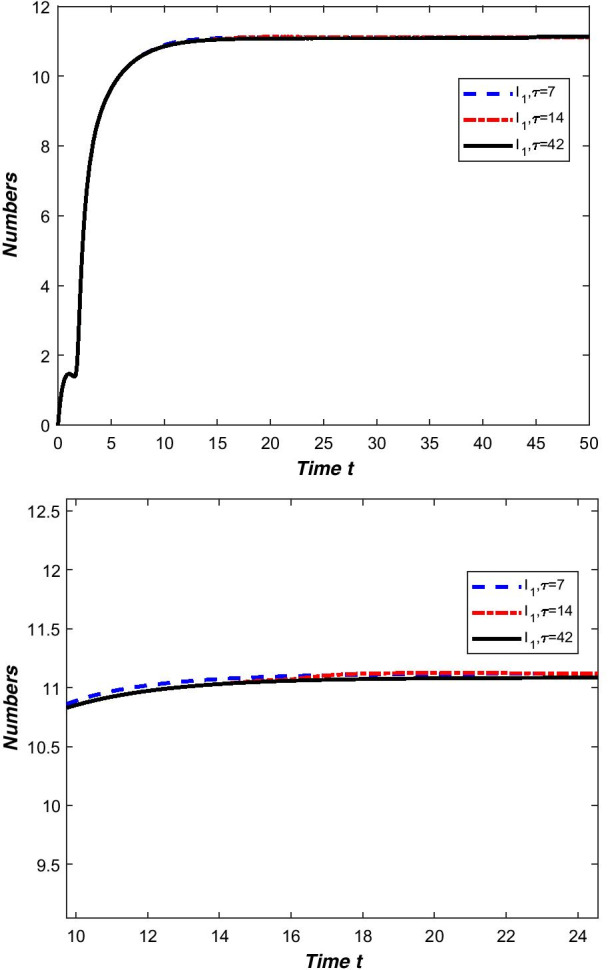
Comparison chart of $\tau =7, \tau =14$ and $\tau =42$

Everyone knows that the COVID-19 epidemic usually has an incubation period of 7–14 days, and there have been previous reports claiming an extremely long incubation period of 42 days. We simulate the changes in the number of people in compartment $I_1$ with a time delay of 7 days, 14 days and 42 days. Because the time delay is relatively short, the changing trends of the three curves are relatively close on the surface. However, we can still clearly see from the first image of Fig. 12 that the three curves are not completely coincident. In order to be able to see the relationship between the three curves clearly, we have also partially enlarged the simulated image (second image of Fig. 12).

From Fig. 12, we can see that when the time delay is equal to 14 days, the number of infected people is the largest in the steady state. Similarly, we also simulate the changes in the number of people in compartment $I_1$ with a time delay of 0 days, 7 days and 14 days. In particular, when $\tau =0$, the original model becomes a new COVID-19 model without time delay.

**Fig. 13:**
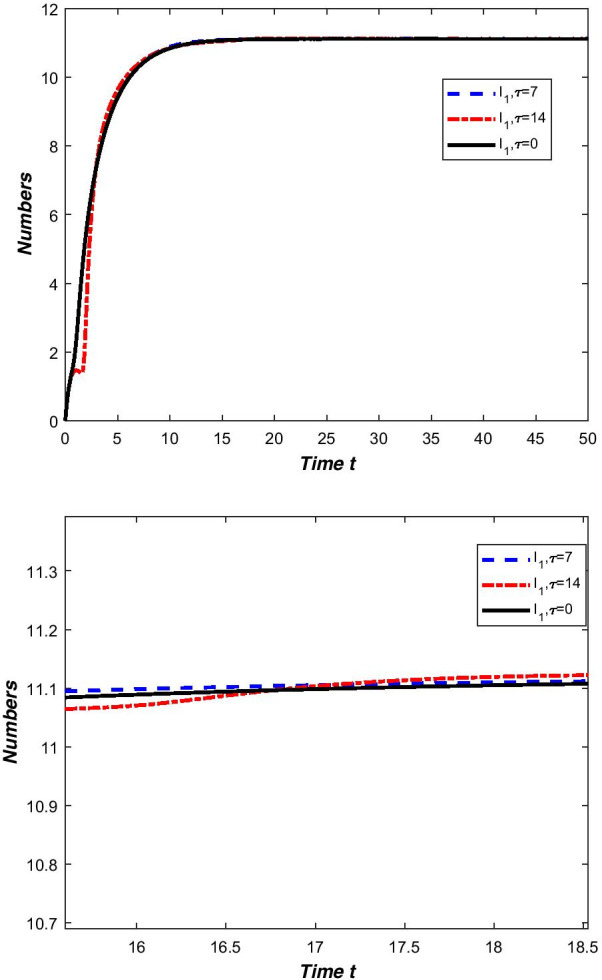
Comparison chart of $\tau =7, \tau =14$ and $\tau =0$

From the simulation results (Fig. 13), we can see that the incubation period of 14 days is still the most serious situation of the epidemic. Therefore, the 14th day of the incubation period is the peak of the possible outbreak of COVID-19. From the perspective of public health, the time delay effect provides the government and medical departments with valuable time for prevention and control deployment. During this period of time, relevant departments can detect asymptomatic infections in a timely manner through effective detection methods and control the spread of the epidemic in local areas. In this way, the time delay period can be described as the golden period for epidemic prevention, control and treatment. Combining Figs. 12 and 13, since the outbreak of the epidemic will weaken after 14 days, the prevention and control of the first 14 days is particularly important. The 14-day quarantine policy introduced by many countries during the COVID-19 epidemic is reasonable. Strict implementation of the relevant policies of 14-day home quarantine has been effective in preventing and controlling the COVID-19 epidemic. If the patient takes targeted self-limiting treatment during the 14-day prime time, such as reducing going out, strengthening exercise, vaccination, oxygen therapy, etc., it can speed up the recovery of the disease.

## Conclusion

Novel coronavirus pneumonia is a self-limiting disease, and targeted self-limiting treatment can speed up the recovery of infected people. This conclusion was questioned at the beginning of the outbreak, but with the accumulation of global experience in treating novel coronavirus pneumonia, such doubts no longer exist. This paper studies the long-term dynamics of the self-limiting time delay diffusion novel coronavirus pneumonia model in a temporal-spatial heterogeneity environment. Through mathematical modeling and rigorous mathematical reasoning, we have proved that targeted self-limiting treatment can effectively control the spread and diffusion of the novel coronavirus pneumonia epidemic. In addition, due to the introduction of temporal-spatial heterogeneity environment in the model, the proof of the global stability of the model is much more difficult than that of the constant coefficient model. In this proof process, we found that the principal eigenvalue of the system can be used as a new threshold to better characterize the epidemic infection ability in a temporal-spatial heterogeneity environment. Furthermore, we used the global attractor method to discuss the global stability and global exponential attractivity of the spread of novel coronavirus pneumonia in a temporal-spatial heterogeneous environment. With the help of numerical simulations, we intuitively demonstrated the impact of the temporal-spatial heterogeneity environment on the spread of the novel coronavirus pneumonia epidemic and the promotion of self-limiting treatment on the prevention and control of the novel coronavirus pneumonia epidemic. Numerical simulation results show that the spread of the global novel coronavirus pneumonia epidemic has fluctuated and increased due to seasonal changes and regional differences, and increasing the proportion of self-limiting treatment can greatly reduce the number of infected people. At the same time, time delay also plays a very important role in the spread of the epidemic. The 14th day is the peak of a concentrated outbreak of infected people.

## Data Availability

The data in this article are all public data published on the official websites of the World Health Organization, the Chinese Health Commission, Centers for Disease Control and Prevention, and everyone can check them on the corresponding websites.

## Abbreviation

WHO: World Health Organization
