# Correction to: The effect of self-limiting on the prevention and control of the diffuse COVID-19 epidemic with delayed and temporal-spatial heterogeneous

**DOI:** 10.1186/s12879-021-06875-1

**Published:** 2022-01-05

**Authors:** Cheng-Cheng Zhu, Jiang Zhu

**Affiliations:** 1 School of Science, Jiangnan University, Wuxi, 214122 China; 2 School of Mathematics and Statistics, Jiangsu Normal University, Xuzhou, 221116 China

## Correction to: BMC Infect Dis (2021) 21:1145 https://doi.org/10.1186/s12879-021-06670-y

Following publication of the original article [1], an error was identified in page 11.

The font of the first and fifth lines currently read: L

The font should read: ${\mathcal{L}}.$

Furthermore an error was identified in formula (7).

The formula currently read: $\mu \left( {x,t} \right)A.$

The formula should read $\Lambda \left( {x,t} \right).$

The original article [1] has been corrected.
